# Large-Scale Analysis of B-Cell Epitopes on Influenza Virus Hemagglutinin – Implications for Cross-Reactivity of Neutralizing Antibodies

**DOI:** 10.3389/fimmu.2014.00038

**Published:** 2014-02-07

**Authors:** Jing Sun, Ulrich J. Kudahl, Christian Simon, Zhiwei Cao, Ellis L. Reinherz, Vladimir Brusic

**Affiliations:** ^1^Cancer Vaccine Center, Dana-Farber Cancer Institute, Harvard Medical School, Boston, MA, USA; ^2^Department of Medicine, Harvard Medical School, Boston, MA, USA; ^3^Center for Biological Sequence Analysis, Technical University of Denmark, Lyngby, Denmark; ^4^School of Life Sciences and Technology, Tongji University, Shanghai, China; ^5^Laboratory of Immunobiology, Department of Medical Oncology, Dana-Farber Cancer Institute, Harvard Medical School, Boston, MA, USA

**Keywords:** influenza virus, neutralizing antibodies, B-cell epitope, cross-reactivity, discontinuous peptide

## Abstract

Influenza viruses continue to cause substantial morbidity and mortality worldwide. Fast gene mutation on surface proteins of influenza virus result in increasing resistance to current vaccines and available antiviral drugs. Broadly neutralizing antibodies (bnAbs) represent targets for prophylactic and therapeutic treatments of influenza. We performed a systematic bioinformatics study of cross-reactivity of neutralizing antibodies (nAbs) against influenza virus surface glycoprotein hemagglutinin (HA). This study utilized the available crystal structures of HA complexed with the antibodies for the analysis of tens of thousands of HA sequences. The detailed description of B-cell epitopes, measurement of epitope area similarity among different strains, and estimation of antibody neutralizing coverage provide insights into cross-reactivity status of existing nAbs against influenza virus. We have developed a method to assess the likely cross-reactivity potential of bnAbs for influenza strains, either newly emerged or existing. Our method catalogs influenza strains by a new concept named discontinuous peptide, and then provide assessment of cross-reactivity. Potentially cross-reactive strains are those that share 100% identity with experimentally verified neutralized strains. By cataloging influenza strains and their B-cell epitopes for known bnAbs, our method provides guidance for selection of representative strains for further experimental design. The knowledge of sequences, their B-cell epitopes, and differences between historical influenza strains, we enhance our preparedness and the ability to respond to the emerging pandemic threats.

## Introduction

Influenza epidemics result in substantial morbidity and mortality (1). The World Health Organization (WHO) Global Influenza Network provides annual recommendations on antigenic variants to be included in the influenza vaccine formulations. Influenza virus has low-fidelity polymerases that result in high mutation rates (2). As a consequence, seasonal influenza viruses efficiently escape from acquired immunity in the human population through antigenic drift increasing the impact of seasonal influenza. The antigenic shift in influenza A viruses – the reassortment of multiple viral genomes resulting in new strains with recombined antigens – leads to occasional worldwide pandemics that result in significant morbidity and, usually, high mortality. High transmissibility of influenza combined with rapid mutation rates makes the discovery of novel influenza therapeutics an imperative (3). The main challenge in developing antibody-based prophylactics and therapeutic vaccine against influenza is to understand the variation generated by the virus and developing means to elicit broadly neutralizing antibody responses.

The majority of neutralizing antibodies (nAbs) generated during a normal immune response target hemagglutinin (HA) and block viral entry into host cells (4). However, significant sequence diversity among HA genes limits the protective breadth of these nAbs (5). This sequence diversity of influenza A virus is high – there are 17 HA serotypes that belong into two major groups called group 1 (Grp1: H1, H2, H5, H6, H8, H9, H11, H12, H13, H16, and H17), and group 2 (Grp2: H3, H4, H7, H10, H14, and H15) (6). C179, the first neutralizing antibody reported to neutralize strains from H1 and H2 of influenza A virus, was isolated from mice immunized with the A/Okuda/57 (H2N2) strain (7). Later it was found that C179 was able to cross-neutralize H1, H2, H5, H6, and H9 subtypes (8–11). The next major advance in the field came about 15 years later (12), a novel class of human antibodies encoded by the V_H_1–69 gene were discovered. Among these antibodies, a series of broadly neutralizing antibodies (bnAbs) have been described, such as CR6261 and F10 (13). Most bnAbs that neutralize influenza A virus have been reported to neutralize strains from either exclusively Grp1 or Grp2. FI6v3 (14) and 39.29 (5) are the only antibodies reported to neutralize human influenza isolates from both Grp1 and Grp2. Influenza B viruses are classified within a single influenza type, with two antigenically and genetically distinct lineages that co-circulate (15), represented by the prototype viruses B/Victoria/2/1987 (Victoria lineage) and B/Yamagata/16/1988 (Yamagata lineage) (16). Antibody CR8071 (17) is a bnAb against influenza B viruses, with neutralizing ability for both Victoria and Yamagata lineages. bnAb CR9114 (17) binds a conserved epitope on the HA stem and was shown to neutralize all tested influenza A viruses. However, it did not show *in vitro* neutralizing activity against influenza B viruses at the tested concentrations (17).

Generally, the neutralizing effectiveness of these bnAbs was evaluated using representative strains from the subtypes of influenza A virus or lineages of influenza B virus. Because of the high variability of HA genes, such evaluation might result in a conclusion that is limited to the tested viral variants. To determine the landscape of nAbs and better understand their cross-reactivity properties, we performed a systematic study of B-cell epitopes of a selection of nAbs against influenza virus. Antibodies recognize discrete sites on the surface of macromolecule called B-cell epitopes (antigenic determinants). Some 10% of B-cell epitopes are linear peptides while 90% are formed from discontinuous amino acids that create surface patches through the three dimensional (3D) conformation of proteins (18). We defined a novel way of describing discontinuous motifs, using virtual peptides, to represent B-cell epitopes and further used this representation to estimate potential cross-reactivity and neutralizing coverage of these nAbs.

Functional characterization of the increasing number of nAbs and known crystal structures of these nAbs complexed with HA proteins enables us to precisely define their B-cell epitopes. A large number of sequences of influenza variants are available in public databases (19) enabling systematic bioinformatics analysis of cross-reactivity of nAbs against influenza virus. Such systematic analysis improves our understanding of antibody/antigen interactions, facilitates mapping of the known universe of target antigens, and allows the prediction of cross-reactivity. These methods and tools are useful for the design of broadly protective vaccines against emerging pathogens. This article describes a study of influenza HA cross-reactivity, but the method is applicable to any viral pathogen where information about nAbs and a collection of variant sequences of the target antigen are available.

## Materials and Methods

### Neutralizing antibodies against hemagglutinin

The names and specificities of nAb against influenza virus HA were collected from published papers. Twenty-two nAbs against influenza virus with crystal structures available in PDB were collected from published articles (Table 1). Fifteen of these nAbs target at the globular head of HA, and for the other seven, the binding sites are located on HA stem region.

**Table 1:** **Summary of well-characterized neutralizing antibodies against influenza virus**.

Location	Neutralizing antibodies	PDB ID	Neutralizing breadth	Reference
Head	1F1	4GXU	H1	(20)
	2D1	3LZF	H1	(21)
	2G1	4HG4	H2	(17)
	8F8	4HF5	H2	(17)
	8M2	4HFU	H2	(17)
	*BH151*	*1EO8*	*A/X-31 (H3N2)*	(22)
	C05	4FQR	H1, H2, H3, H9	(23)
	CH65	3SM5	H1	(24)
	CH67	4HKX	H1	(25)
	CR8059	4FQK	Influenza B virus	(17)
	CR8071	4FQJ	Influenza B virus: Yamagata and Victoria	(17)
	*HC19*	*2VIR*	*A/X-31 (H3N2)*	(26)
	*HC45*	*1QFU*	*A/X-31 (H3N2)*	(27)
	*HC63*	*1KEN*	*A/X-31 (H3N2)*	(28)
	S139/1	4GMS	H1, H2, H3, H13, H16	(8, 29)
Stem	39.29	4KVN	H1, H2, H3	(5)
	C179	4HLZ	Grp1: H1, H2, H5, H6, H9	(30)
	CR6261	3GBN/3GBM	Grp1: H1, H2, H5, H9	(31, 32)
	CR8020	3SDY	Grp2: H3, H7, H10	(33)
	CR9114	4FQI/4FQV/4FQY	Grp1: H1, H2, H5, H6, H8, H9, H12	(17)
			Grp2: H3, H4, H7, H10	
	F10	3FKU	Grp1: H1, H2, H5, H6, H8, H9, H11	(13)
	FI6v3	3ZTJ/3ZTN	H1, H3, H5, H7	(14)

**Figure 1:**
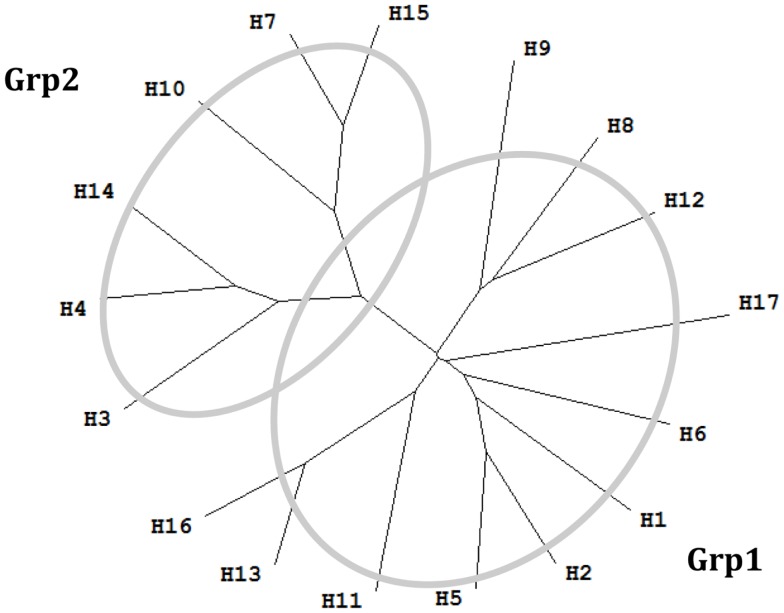
**Phylogenetic tree of 17 influenza A virus subtypes**. Representative sequences were selected for each subtype (34) and the phylogenetic tree was made with ClustalX (35) and TreeView (36). H1: A/California/04/2009(H1N1); H2: A/Singapore/1/1957(H2N2); H3: A/Aichi/2/1968(H3N2); H4: A/duck/Czechoslovakia/1956(H4N6); H5: A/VietNam/1203/2004(H5N1); H6: A/chicken/California/431/2000(H6N2); H7: A/Turkey/Italy/8458/2002(H7N3); H8: A/Turkey/Ontario/6118/1968(H8N4); H9: A/Swine/Hong Kong/9/98(H9N2); H10: A/chicken/Germany/N/1949(H10N7); H11: A/duck/England/1/1956(H11N6); H12: A/duck/Alberta/60/1976(H12N5); H13: A/gull/Maryland/704/1977(H13N6); H14: A/Mallard/Astrakhan/263/1982(H14N5); H15: A/shearwater/West Australia/2576/79(H15N9); H16: A/black-headed gull/Sweden/2/99(H16N3); and H17: A/little yellow-shouldered bat/Guatemala/060/2010 (H17N10).

*The nAbs in underlined italics are nAbs specific for strain A/X-31 (H3N2). The designation of two groups (Grp1 and Grp2) of influenza A virus subtypes are shown in Figure 1*.

The majority of these nAbs were observed to bind or neutralize influenza A virus isolated either from Grp1 or Grp2. Antibodies FI6v3, CR9114, and 39.29 were shown to neutralize influenza strains within both Grp1 and Grp2 (5, 14, 27). Antibodies CR8059 and CR8071 (17) were the only two nAbs for influenza B virus. CR8059 is a light chain D95aN variant of CR8071. Since the mutation on CR8059 is not present at the binding interface and does not affect the binding, only CR8071 was used in the following study (17). The majority of these nAbs were shown to neutralize more than one strain, some of them are broadly neutralizing across subtypes of influenza A virus or lineages of influenza B virus. The Abs BH151, HC19, HC45, and HC63 were shown to specifically neutralize HA from the A/X-31(H3N2) strain. The available structures of nAb/HA complexes were downloaded from PDB (37).

### Validated influenza strains by neutralizing antibodies

Binding and neutralization assays were collected from published materials. Binding and non-binding strains were classified according to their affinity measurements. The thresholds used to discriminate binding and non-binding strains were inconsistent in different studies: the lowest affinity detectable values were set as 10^−4^ M (17), 10^−5^ M (33), and ~10^−6^ M (20). In some reports, nAbs showed positive binding results but did not display neutralization ability to the same strains [e.g., nAb CR9114 against strain B/Florida/4/2006 (Yamagata) (17)]. Because of the lack of standardized thresholds and ambiguous definition of binding, only results that indicate non-binding of antibodies were considered as useful information and were retained for the subsequent analysis as negatives.

The neutralized and the escape strains were detected using the microneutralization assay (38) or HA inhibition assay (39). Several measurements were suggested in these studies:

1. The lowest concentration of nAb that displayed inhibition of hemagglutination or microneutralizing activity were set as either 2.5 μg/mL (40) or 5 μg/mL (41).
2. The 50% inhibitory concentration was set to IC_50_ = 50 μg/mL (17).
3. The effective concentration of antibody needed to inhibit at least 99% of viral infectivity was set as EC_99_ = 100 μg/mL (24, 25).

The HA sequences of strains that were experimentally validated for neutralization by studied antibodies (“validated strains”) were retrieved from the literature. The influenza strains HA sequences were collected from the literature or, if absent, from the Influenza Knowledge Base (FLUKB)^1^. All experimentally validated strains were grouped into either neutralized strains or escape strains. The neutralized strains were selected based on reported experimental evidence. The escape strains included true escape strains as well as strains that were reported not to bind nAbs. We did not find any discrepancies in reported neutralizing properties across different studies used to collect functional data.

### Hemagglutinin sequences

All HA sequences were downloaded from the Influenza Knowledge Base (FLUKB^1^, dated August 26th, 2013). After removing the incomplete sequences (fragments), 45,812 full-length HA sequences were left in the data set (HA sequence dataset) for further analysis.

### Generation of multiple sequence alignment of hemagglutinin sequences

The HA sequences of influenza strains from FLUKB were aligned using the MAFFT tool (42). The resulting multiple sequence alignment (MSA) results provided a consistent numbering scheme for all the further analyses. MSA were generated for both experimentally validated strains of HA and for all entries from FLUKB. For each nAb, every HA sequence from the crystal structure and from the experimentally validated strains were searched individually within the FLUKB database to find a strain with highest similarity using BLAST (43). This procedure was done to ensure that residue position mapping in following steps is consistent with the numbering scheme.

### Identification of B-cell epitopes

B-cell epitope were identified from antigen–antibody structure, using a formula with the combination of the measurements of accessible surface area (ASA) and atom distance. For each residue from HA antigen, the ASA value was calculated using Naccess software (44) for both free HA and for HA coupled with an antibody. Residues *r_i_* with ASA loss more than 20% were selected as epitope residues,

$$r_{i}\in \left\{\mathrm{epitope residues}\right\}\mathrm{if}\;\frac{{\mathrm{ASA}}_{\mathrm{free}}-{\mathrm{ASA}}_{\mathrm{coupled}}}{{\mathrm{ASA}}_{\mathrm{free}}}>0.2.$$

The majority of contacts between two contacting atoms occur at distance smaller than 5 Å separation (45). Euclidean distance was calculated between atoms *a_i_* and *a_j_* using their coordinates *a_i_*(*x_i_, y_i_, z_i_*) and *a_j_*(*x_j_, y_j_, z_j_*) in PDB structure data,

$$d_{ij}=\sqrt{{\left(x_{i}-x_{j}\right)}^{2}+{\left(y_{i}-y_{j}\right)}^{2}+{\left(z_{i}-z_{j}\right)}^{2}}.$$

Hemagglutinin residues *r_i_* whose minimum atom distance to the closest nAb atom was within 4 Å were also incorporated in the epitope. The minimal atom distance was defined as:

$$\begin{array}{llll} & d_{\min}\;=\;\min\left\{d_{ij}\right\},\;a_{i}\;\in \;\mathrm{antigen residue}\;r_{i},a_{j}\;\in \;\mathrm{antibody residue}\;r_{j},\; & & \;\\ & \;r_{i}\in \left\{\mathrm{epitope residue}\right\}\;\mathrm{if}\;d_{\min}<4Å.\; & & \; \end{array}$$

The residues that satisfy either of these two conditions (ASA loss or minimum distance) are considered to constitute a B-cell epitope.

The specific residues on HA that form hydrogen bonds, salt bridges, disulfide bonds, and covalent bonds between the HA and nAb were considered to define a B-cell epitope. The antigen/antibody interaction was further analyzed using PISA tool (46). The analysis of HA structures showed that all the hydrogen bonds, salt bridges, disulfide bonds, and covalent bonds between HA and nAb in each studied structure were incorporated in B-cell epitopes defined in the previous step.

### Extraction of discontinuous motifs from validated strains

For each nAb, using the MSA result and the standardized numbering, the residue positions of B-cell epitope identified from the HA/antibody crystal structure were mapped onto all HA sequence of validated strains. Then discontinuous motifs composed of mapped residues were extracted from these sequences. These discontinuous motifs were classified as either “neutralized” or “escape” motifs according to the experimental validation status of the corresponding strain.

### Mapping of discontinuous motifs to HA sequence dataset

For each nAb, based on the MSA result, the residue positions of B-cell epitope identified from the HA/antibody crystal structure were mapped onto the HA sequence dataset. A “discontinuous peptide” composed of amino acids that form B-cell epitope, in order that they appear in the sequence, was extracted from each HA sequence. By comparing the discontinuous peptides to all validated neutralized and escape motifs from experimentally validated strains, each discontinuous peptide was classified as neutralized (if 100% matching a neutralized epitope motif), escape (if 100% matching an escape epitope motif), or non-validated (if 100% matching validation data are missing). The term “discontinuous motif” indicates positions that define each B-cell epitope extracted from experimentally validated strains collected from publications, while term “discontinuous peptide” represents specific B-cell epitopes extracted from the HA sequence dataset.

## Results

### B-cell epitope regions

**Figure 2:**
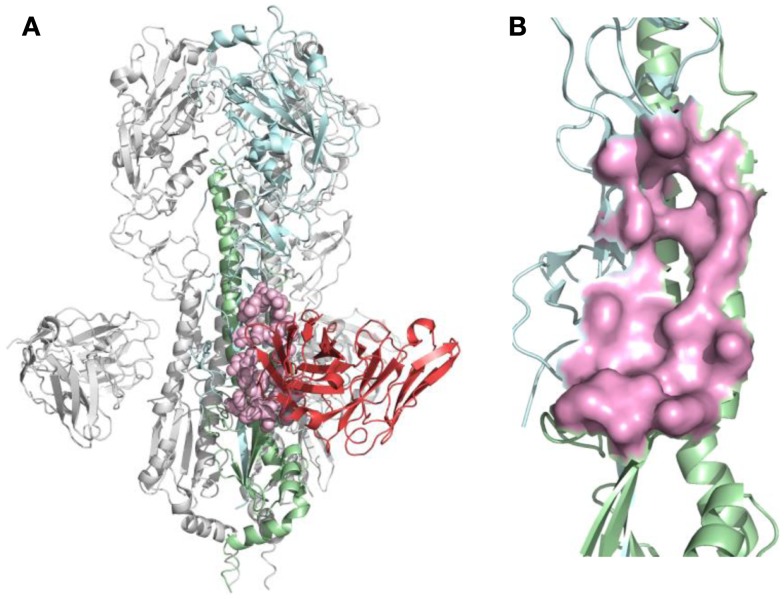
**B-cell epitope on the structure of neutralizing antibody F10 binding HA protein (PDB ID: 3FKU)**. **(A)** Complex of F10-HA [A/Vietnam/1203/04(H5N1)]. The structure is a HA trimer of three identical copies (one of them is colored as cyan and green; the other two are in gray). Each copy contains the HA1 (cyan) and HA2 (green) chain, also the heavy chain of F10 (red), the neutralized epitope is highlighted in pink; **(B)** Close-up view of neutralized epitope identified on the structure (highlighted as pink surface).

For each nAb, the B-cell epitope was identified from the crystal structure as described in Section “Materials and Methods.” The structure of nAb F10-H5 (13) and identified epitope are illustrated in Figure 2. After B-cell epitopes of all studied nAbs were mapped to the same template structure, the overlapping of binding sites were found among different nAbs, particularly at the receptor-binding site (RBS), which is the necessary structure for binding to the sialic acid receptors during virus infection.

**Figure 3:**
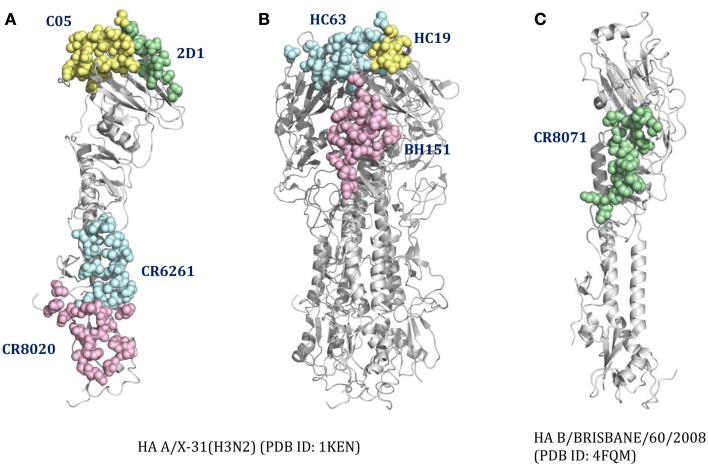
**The distinct B-cell epitope regions recognized by representative nAbs**. The B-cell epitope regions of **(A)** represent cross-reactive nAbs against influenza A virus; **(B)** represent strain-specific nAbs against X-31(H3N2); **(C)** represent broadly nAb CR8071 against influenza B virus. The epitope regions of nAbs target influenza A virus were mapped on the monomer **(A)** or trimer **(B)** HA from A/X-31(H3N2) (PDB ID: 1KEN). The structure of B/Brisbane/60/2008 HA (PDB ID: 4FQM) was used as a template structure for influenza B virus. Different colors here were used for distinguishing B-cell epitope regions.

For cross-reactive nAbs against influenza A virus, four major binding locations on HA structure are apparent: two of them reside on the globular head of HA and the other two target the stem region of HA (Table 2; Figure 3). The RBS is a heavily targeted area, with overlapping epitopes defined by eight nAbs. The only nAb that binds HA head but not the RBS is 2D1 (21). The 2D1 recognizes the Sa site of A/South Carolina/1/1918(H1N1). Sa site is one of the earliest known antigenic sites (47), which is proximal to the receptor-binding pocket. The detailed comparison of epitope residue positions between 2D1 and the other HA head-targeted nAbs are listed in Table 3. In contrast to the Abs that interact with the HA head, a series of nAbs recognize another highly conserved helical region in the membrane-proximal HA stem. The epitopes on F subdomain (CR6261, 39.29, etc.) and stem base (CR8020) are adjacent to each other, with a small number of shared residues. The only broadly nAb neutralizing influenza B virus, CR8071 binds to the lower region of the globular head of HA – the “head base” (Figure 3C). All the remaining antibodies analyzed in our study bind specifically the HA on A/X-31(H3N2) strain. All X-31 specific nAbs complex with the membrane-distal domain of HA. NAbs BH151 and HC45 (22) recognize a single epitope located at the base of the eight-stranded antiparallel β-sheet structure. The HC19 binding site is adjacent to the RBS. The HC63 epitope shares several residues with HC19, thereby the antibody binding site overlaps the membrane-distal domains of two HA monomers.

**Table 2:** **B-cell epitope regions of the 22 neutralizing antibodies**.

Binding site	Influenza A virus				Influenza B virus
	Sa site	Near RBS	F subdomain	Stem base	Head base
**CROSS-REACTIVE NEUTRALIZING ANTIBODIES**					
**nAbs**	**2D1**	**C05**	**CR6261**	**CR8020**	**CR8071**
		1F1	39.29		CR8059
		2G1	C179		
		8F8	CR9114		
		8M2	F10		
		CH65	FI6v3		
		CH67			
		S139/1			

**Binding site**	**Head base**	**RBS**	**Near RBS**	

**X-31-SPECIFIC NEUTRALIZING ANTIBODIES**					
**nAbs**	**BH151**	**HC19**	**HC63**		
	HC45				

*The nAbs are classified as cross-reactive or X-31-specific. For each binding region, a representative nAb was selected (shown in bold) and its B-cell epitope was mapped on the structures shown in Figure 3*.

**Table 3:** **B-cell epitope overlap for nAbs targeting HA head region**.

	Sa	Near RBS									Sa	Near RBS							
	2D1	1F1	2G1	8F8	8M2	C05	CH65	CH67	S139/1		2D1	1F1	2G1	8F8	8M2	C05	CH65	CH67	S139/1
A98	−	+	−	+	+	+	+	+	+	A163	+	−	−	−	−	−	−	−	−
A125	+	−	−	−	−	−	−	−	−	A165	+	−	−	−	−	−	−	−	−
A126	+	−	−	−	−	−	−	−	−	A166	+	−	−	−	−	−	−	−	−
A128	+	−	+	−	−	−	−	−	−	A167	+	−	−	−	−	−	−	−	−
A130	+	−	+	+	+	−	−	+	−	A169	+	−	−	−	−	−	−	−	−
A131	−	−	−	−	−	+	−	−	+	A183	−	+	−	−	−	+	+	−	+
A132	−	−	+	+	+	−	−	−	−	A185	−	+	−	−	−	−	−	−	−
A133	−	+	+	+	+	+	−	−	−	A186	−	+	−	−	+	+	−	−	+
A134	−	−	+	+	+	+	+	+	+	A187	−	+	−	+	+	+	+	+	−
A135	−	+	−	−	−	+	+	+	+	A188	−	−	−	−	+	−	−	−	−
A136	−	−	+	+	+	+	+	+	+	A189	−	+	−	+	+	+	+	+	+
A137	−	−	+	+	+	+	+	+	+	A190	−	+	+	+	+	+	+	+	+
A140	−	−	−	+	−	−	−	−	−	A192	−	+	−	−	+	+	+	+	+
A143	−	−	−	+	−	−	−	−	−	A193	−	+	+	+	+	+	+	+	+
A144	−	−	−	+	−	−	−	−	−	A194	−	+	+	+	+	+	+	+	+
A145	−		+	+	+	+	−	−	+	A196	−	+	−	−	−	−	+	+	+
A153	−	+	+	+	+	+	+	+	+	A197	+	−	−	−	−	−	−	−	−
A155	−	+	+	+	+	+	+	+	+	A219	−	+	−	−	−	−	−	+	−
A156	−	+	+	+	+	+	+	+	+	A222	−	+	−	−	+	−	+	−	−
A157	+	−	+	−	−	−	−	−	+	A225	−	+	−	−	+	+	+	+	+
A158	+	−	+	+	+	+	+	+	+	A226	−	+	−	+	+	+	+	+	+
A159	+	+	+	+	+	+	+	+	+	A227	−	+	−	−	+	+	+	−	−
A160	+	−	−	−	−	−	+	+	+	A228	−	+	−	−	+	+	−	−	+
A161	+	−	−	−	−	−	−	−	−	A246	+	−	−	−	−	−	−	−	−
A162	+	−	−	−	−	−	−	−	−	A248	+	−	−	−	−	−	−	−	−

*The epitope residue positions of nine nAbs were mapped to the 1EO8 structure chain A. The symbol “+” indicates a contact epitope residue by corresponding nAb, and the symbol “−” means it is not a epitope position. 2D1, with a different epitope area to other eight nAbs, is labeled in red*.

### Experimentally validated discontinuous motifs

**Figure 4:**
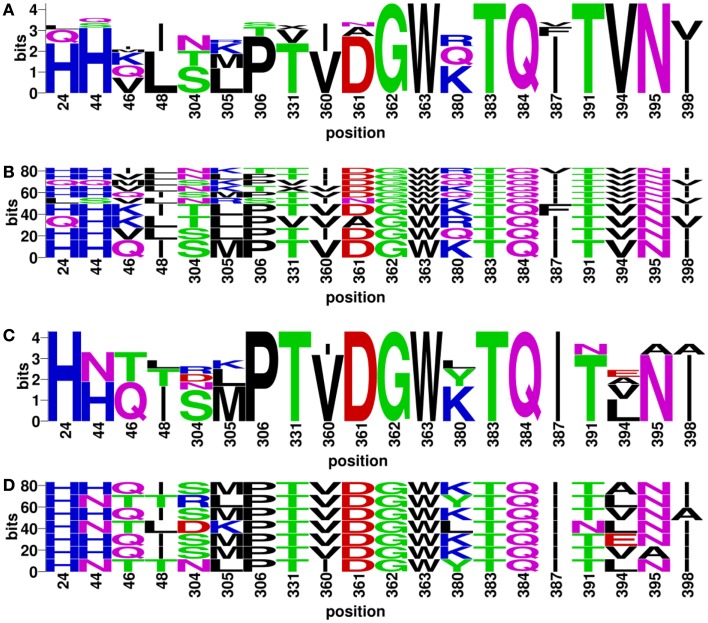
**Neutralized and escape discontinuous motifs from experimentally validated sequences with nAb F10**. The WebLogo shows global **(A)** neutralized motifs, and **(C)** escape motifs and BlockLogo shows individual **(B)** neutralized motifs, and **(D)** escape motifs. The extracted discontinuous motif extracted from the structure (PDB ID: 3FKU, chain A and B), corresponds to the positions of reference sequence [FLU0293715, A/Viet Nam/1203/2004(H5N1)]: 24, 44, 46, 48, 304, 305, 306, 331, 360, 361, 362, 363, 380, 383, 384, 387, 391, 394, 395, and 398.

Discontinuous motifs were extracted from the validated sequences as described in Section “Materials and Methods,” and presented by WebLogo (48) and BlockLogo^2^ [Ref. (49)]. WebLogo figures consist of stacks of amino acids, while the overall height of the stack indicates the sequence conservation at that position, and the height of symbols within the stack indicates the relative frequency of each amino or nucleic acid at that position. While BlockLogo is a web-based application for visualization of protein and nucleotide fragments, continuous protein sequence motifs, and discontinuous sequence motifs using calculation of block entropy from MSAs. The BlockLogo figures present the actual combinations of amino acids, and the height of each combination represents its relative frequency. In the nAb F10 as an example, the neutralized and escape discontinuous motifs are shown in Figures 4A,C (WebLogo figures), and Figures 4B,D (BlockLogo figures). WebLogos show a clear overall description of each residue conservation difference between individual neutralized and escape motifs. For example, 44N, 48T, 304R/D, 380L/Y, 391N, 394E/A/L on F10 epitope region are likely to contribute to the escape strains. In the BlockLogo figures, specific neutralized and escape B-cell epitopes of F10 were listed with their frequencies, which can be used for their direct comparison.

### Analysis of variation of discontinuous peptides in HA sequences dataset

For each nAb, the residue positions of their B-cell epitopes were mapped on the complete HA sequences dataset collected from the FLUKB. Amino acid strings representing discontinuous peptides were extracted from the HA sequence of each strain. The variability of discontinuous peptides and validated discontinuous motif coverage were analyzed for each nAb.

**Figure 5:**
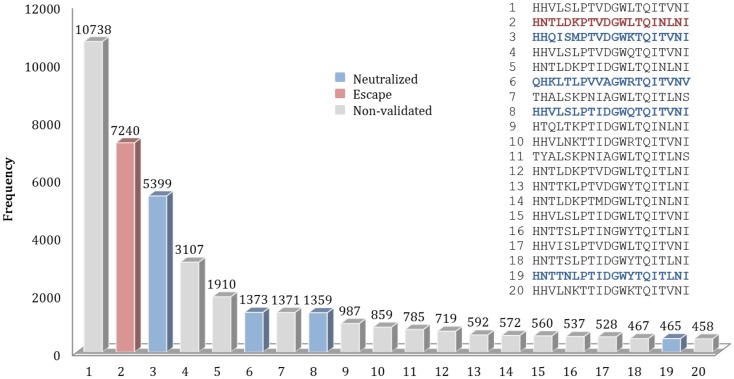
**Frequencies of top 20 discontinuous peptides (B-cell epitope of nAb F10) from the HA sequence dataset**. FLU0243751 (A/Viet Nam/1203/2004) was used as reference HA sequence in the analysis of F10 B-cell epitopes. The corresponding positions of discontinuous peptides on FLU0243751 are: 24, 44, 46, 48, 304, 305, 306, 331, 360, 361, 362, 363, 380, 383, 384, 387, 391, 394, 395, and 398. Discontinuous peptides that were identical to neutralized motifs are shown in blue, while those identical to escape motifs are shown in red. The sequences of Top 20 most frequent discontinuous peptides are listed along with their validation status.

For example, for the nAb F10, 589 different patterns of discontinuous peptides were generated among all 45,812 sequences in HA sequence dataset, using the F10 B-cell epitope identified from the crystal structure. In the next step, the discontinuous peptides were sorted according to their frequencies. The second most frequent peptide in FLUKB is identical an escape motif, while the 6th, 8th, and 19th are each identical to one of the neutralized motifs. However, the most frequent F10 discontinuous peptide in FLUKB (see text footnote 1) has not been experimentally tested (Figure 5), along with other 14 discontinuous peptides. The analysis of differences between the most frequent discontinuous peptide and neutralized or escape motifs was inconclusive. Therefore future experimental studies should include a representative sequence containing the discontinuous peptide HHVLSLPTVDGWLTQITVNI that is present in more than 10,000 entries in the FLUKB. We also recommend that motifs 1, 4, 5, 7, 9–18, and 20 are considered for the experimental validation. The remaining sequences are less common, each having <400 sequences in the data set.

The discontinuous peptides were generated and the variability was investigated for all cross-reactive nAbs (Table 4). The B-cell epitope regions on the HA stem are less variable as compared to the epitopes on the HA head. The specific result generated within each subtype in HA sequence dataset show similar patterns as for all subtypes (data not shown). This conclusion is consistent with our previous knowledge that the globular head of HA1 has a higher mutation rate than the stem (29), making the stem a more conserved region for bnAbs targeting.

**Table 4:** **The number of different discontinuous peptides from B-cell epitopes of each nAb in the HA sequence dataset**.

Neutralizing type	Neutralizing epitope regions		Neutralizing antibodies	Number of different discontinuous peptides
Influenza A virus
	Head	Sa site	2D1	2,190
		Near RBS	1F1	2,887
			2G1	2,127
			8F8	2,885
			8M2	3,290
			C05	3,020
			CH65	2,727
			CH67	2,773
			S139/1	3,070
	Stem	F subdomain	39.29	983
			C179	755
			CR6261	658
			CR9114	663
			F10	589
			FI6v3	905
		Stem base	CR8020	620
Influenza B virus
	Head base		CR8071	848

### Discontinuous motifs coverage in HA sequences dataset

**Figure 6:**
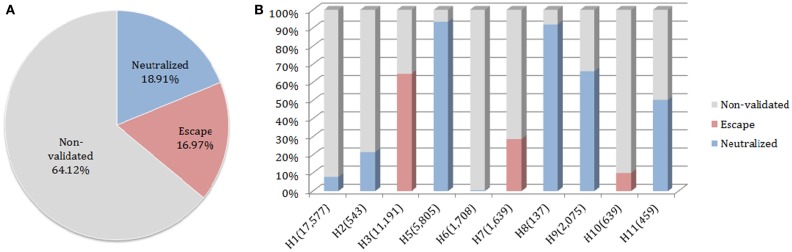
**Motif coverage for nAb F10**. **(A)** The coverage of neutralized and escape discontinuous motifs, and non-validated discontinuous peptides within 45,812 HA sequences extracted from the FLUKB; **(B)** The motif coverage by subtype, the numbers in brackets indicate the number of sequence within the specific subtype (among 45,812 HA sequences).

The neutralized and escape discontinuous motifs of nAb F10 have covered 19 and 17% of FLUKB, respectively, while the discontinuous peptides from 64% of the strains have not been validated (Figure 6A). Viewed by the subtype, F10 neutralized coverage of subtypes H5, H8, H9, and H11 are higher (50–90%) than of H1 and H2 (5–20%), while the coverage of subtype H6 is negligible (8 in 1708 H6 sequences) (Figure 6B).

**Figure 7:**
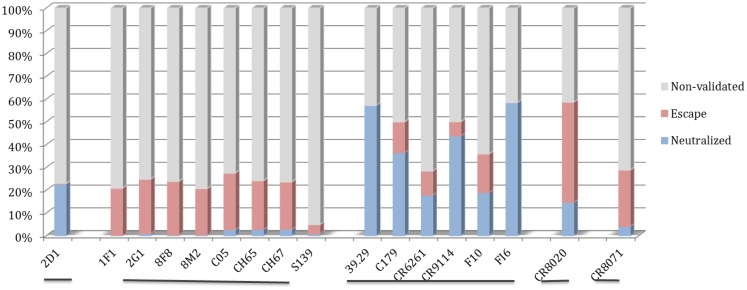
**Discontinuous motif coverage in the HA sequence dataset for cross-reactive nAbs**. Only the coverage data for cross-reactive nAbs are shown here. The nAbs are grouped based on their binding locations and influenza types, from left to right: Sa site, near RBS, F subdomain, stem base on influenza A virus, and head base on influenza B virus.

The motif coverage analysis within the 45,812 HA sequences was performed for all nAbs. For the nAbs with available cross-reactivity data, the motif coverages were different between the nAbs targeting the HA globular head and those targeting the stem part. The nAbs that bind stem normally have higher neutralized motif coverage than those that bind the globular head (Figure 7).

**Figure 8:**
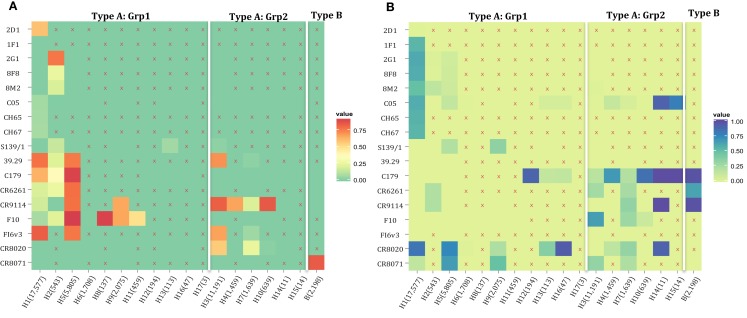
**The neutralized and escape motif coverage on each subtype within FLUKB of neutralizing antibodies**. The heat map of **(A)** neutralized and **(B)** escape coverage are shown. Each cell on the grid represents coverage for a specific antibody (row name) on specific subtype (column name). The subtypes were sorted from Grp1 and Grp2 in influenza A virus, and influenza B virus. Different color schemes were used in order to differentiate neutralizing and escape coverage: from green to red/green to blue indicate rising neutralizing/escape coverage in HA sequence dataset. The boxes with symbol “X” indicate that no experimental validation data were available for this study.

The motif coverage is shown as heat map for each subtype and each nAb (Figure 8). The nAbs (such as CR6261, CR9114, F10, and FI6v3) that target stem region are more cross-reactive – they cover more strains, and also more subtypes of influenza.

### Combining of neutralizing antibodies

For each sequence in the HA sequence dataset, 22 strings (discontinuous peptides) were extracted to represent 22 B-cell epitopes by all nAbs analyzed in this study. The majority (82.62%) of all strains in FLUKB have at least one discontinuous peptide that is identical to the validated neutralized motifs (Table 5). A small number (2.25%) of sequences can be neutralized by as many as seven nAbs.

**Table 5:** **Distribution of the number of neutralizing antibodies that share identical neutralized discontinuous motif with sequences within the HA sequence dataset**.

Number of nAbs	Coverage in 45,812 HA dataset (%)
0	17.38
1	12.45
2	11.68
3	13.39
4	31.38
5	1.59
6	9.89
7	2.25

*For each sequence within the 45,812 HA dataset, the number of nAbs that share identical neutralized motif was counted. The number of nAbs in our panel for any given influenza strain can range from 0 to 7*.

**Figure 9:**
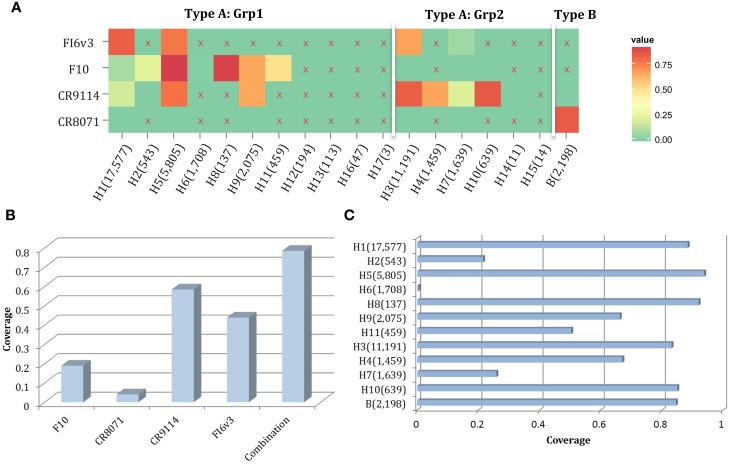
**A combination of four neutralizing subtype-diversified and potent neutralizing antibodies**. **(A)** The heat map of the neutralized result for four nAbs, the color scheme is same as Figure 8A; **(B)** the neutralized coverage of four nAbs individually, and the combination of nAbs on HA dataset; and **(C)** the neutralizing result of combination of four nAbs by subtype. The subtypes were sorted from Grp1 and Grp2 in influenza A virus and influenza B virus. Only subtypes with neutralizing data are shown.

Here, we propose a combination of nAbs, where a small number of nAbs can cover a large proportion of influenza strains. The nAbs FI6v3, F10, CR9114, and CR8071 (Figure 9A) were selected, and the neutralized coverage has increased from 18.91% (F10), 4.06% (CR8071), 43.89% (CR9114), and 58.44% (FI6v3) to 78.45% (Figure 9B) when these antibodies were combined. These nAbs also covered most subtypes of influenza A virus and both lineages in influenza B virus (Figure 9C).

## Discussion

This study presents an overview of binding specificities of reported nAbs, as well as an estimate of their neutralization and escape coverage (neutralization effectiveness) in more than 45,000 HA sequences available in FLUKB. The variety and frequency of discontinuous peptides within different B-cell epitopes have been analyzed in the HA data set. The results of the analysis of discontinuous peptides provide insights into further experimental design: strains with peptides that have high frequency among the strain populations should be given priority for experimental validation and their neutralizing status for specific nAbs.

Of note, additional sequence changes in HA outside the nAb epitope may result in either local or quaternary structural alterations that impacts antibody binding to the epitope *per se*. Likewise, modification of glycosylation sites through sequence change may impact accessibility of antibodies to the neutralization site, creating discordance between sequence identity of binding site shown in BlockLogo and neutralization outcome between two strains of viruses sharing the same epitope sequence. The frequency of such occurrences will be important to determine. Neutralization assays of strains with discontinuous epitopes identical to validated B-cell epitopes will provide a proof of cross-neutralization. Since the experimental validation is time and money consuming, the introduction of extended B-cell epitope (see Supplementary Material) aims to help select representative sequences that differ in extended B-cell epitopes. For each proposed neutralizing or escape peptide (actual B-cell epitope), a small number of variants defined by changes in its environment (extended B-cell epitopes) constitute the majority of strains with the proposed peptide.

On the other hand, before more experimental data generated to fill the existing “non-validated gap,” it will be meaningful to bring out some reasonable estimation. The assumption and methods in this paper are based on complete identity to discontinuous motifs on B-cell epitope (additionally extended B-cell epitope). To check the validity of this assumption, the similarity between discontinuous motifs and discontinuous peptides could be used to estimate and predict neutralization and binding results in the future. For example, a discontinuous peptide with mutated residues of similar feature to the neutralized motif would be considered as “possible neutralized peptide” against specific nAbs. These estimations could also be validated in experimental assays, and then be used to further experimental design iteratively.

## Conclusion

Over the past few years, our understanding of nAbs and their responses against influenza HA have expanded tremendously. Besides the well-known HA head region interactions, an increasing number of characterized nAbs bind and neutralize influenza virus by targeting the more conserved stem regions. Among these stem-targeting nAbs, some show broadly neutralizing ability across subtypes/lineages, even across two groups in influenza A virus strains. However, the related experimental data for majority of nAbs are quite limited.

In sum, we have established a library of validated motifs (extracted from HA sequences in neutralized and escape strains) for each nAb. For any newly emerging strain, the cross-neutralization prediction can be made rapidly for existing nAbs and validation experiments can be designed judiciously. This study provides a method for investigation of cross-reactivity of nAbs against influenza viruses, but is directly applicable to any viral pathogen that has structurally characterized nAbs and a collection of variant sequences of the target antigen. Examples of such pathogens include orthomyxoviruses (influenza); flaviviruses such as dengue or West Nile; arenaviruses such as lymphocytic choriomeningitis virus and human immunodeficiency virus, among others. Insights from such bioinformatics analyses coupled with antibody antigenicity through crystallographic determinations will facilitate electronic neutralization profiling that can be tested empirically in subsequent laboratory neutralization assays.

## Conflict of Interest Statement

The authors declare that the research was conducted in the absence of any commercial or financial relationships that could be construed as a potential conflict of interest.

## Supplementary Material

The Supplementary Material for this article can be found online at http://www.frontiersin.org/Journal/10.3389/fimmu.2014.00038/abstract

## Footnotes

^1^http://research4.dfci.harvard.edu/cvc/flukb

^2^http://research4.dfci.harvard.edu/cvc/blocklogo
