# Supplementary material for: Large-Scale Analysis of B-Cell Epitopes on Influenza Virus Hemagglutinin – Implications for Cross-Reactivity of Neutralizing Antibodies
Source: Front Immunol. 2014 Feb 7;5:38. doi: 10.3389/fimmu.2014.00038 (PMC3916768; doi:10.3389/fimmu.2014.00038)
Supplement: Supplementary file 1 [file 67764_Sun_Presentation1.PDF]

SUPPLEMENTARY FILES

The concept of extended B-cell epitope was introduced here, which was defined based on minimal atom distance as described above (see Material and Method). HA non-epitope (excluding the epitope residues) residues r_i whose minimum atom distance to the closest nAb atom were within (4Å, 6Å] were recruited as extended B-cell epitope for each structure. The extension of B-cell epitope was aimed to investigate the surrounding area, as well as underneath of B-cell epitope.

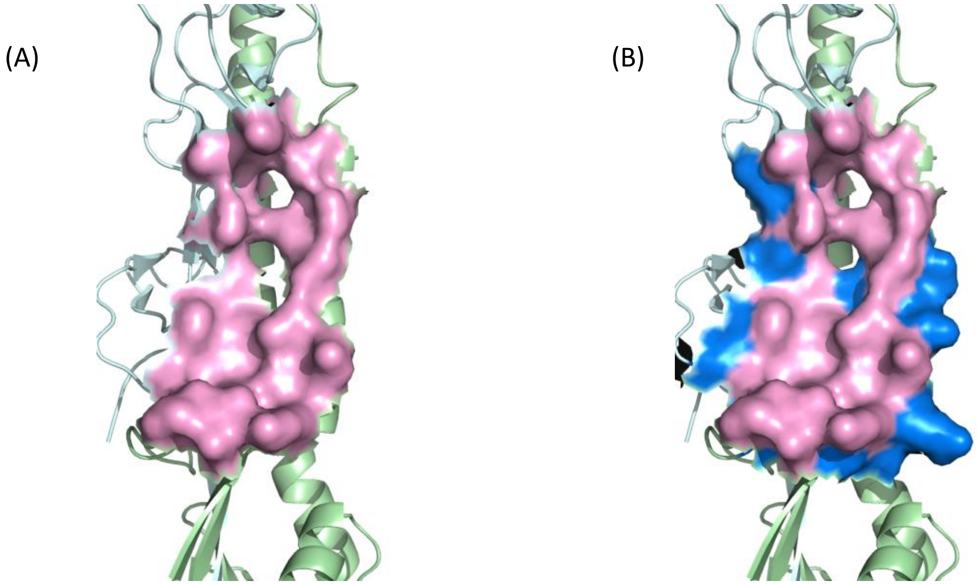

Figure S1. The extension of B-cell epitope. (A) B-cell epitope is highlighted in pink; (B) epitope and extended B-cell epitope is highlighted in blue (non-epitope residue within (4Å, 6Å] to antibody side).

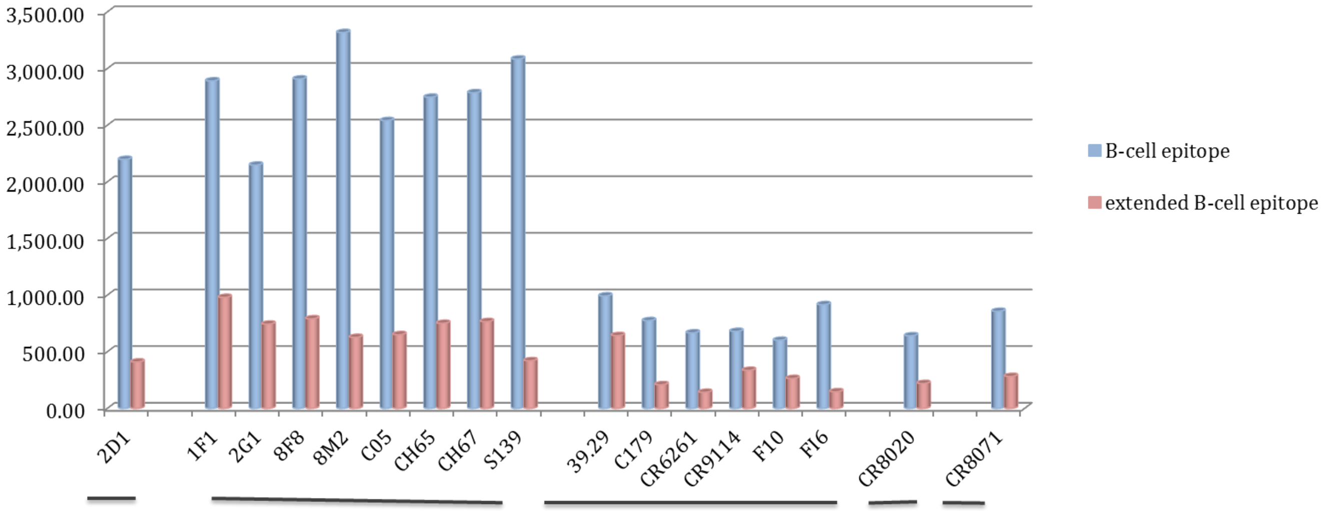

Figure S2. The numbers of different discontinuous peptides from B-cell epitopes and extended B-cell epitopes of each nAb in the HA sequence dataset. The numbers of different B-cell epitopes and extended B-cell epitopes are shown in blue and red bars. The nAbs are grouped based on their binding

locations and influenza types, from left to right: Sa site, near RBS, F subdomain, stem base on influenza A virus, and head base on influenza B virus.

Table S1. The TOP 99 most frequent discontinuous peptides on F10 B-cell epitope region in HA dataset, with the variety of extended F10 B-cell epitope. There are 602 different discontinuous peptides on F10 B-cell epitope region (sorted by frequency) among 45,812 HA sequences. The TOP 99 most frequent discontinuous peptides (≥10 strains with identical discontinuous peptides on B-cell epitope region) are listed here. For each discontinuous peptide, its surrounding discontinuous peptides were also listed with frequencies. The surrounding residues were defined as residues with a distance to antibody between (4Å, 6Å]. The discontinuous peptides identical to validated strains both on B-cell epitope and extended B-cell epitope regions are marked.

TOP 1	HHVLSLPTVDGWLTQITVNI		10738	
	NTSNMADKDIN	10614		
	NTSDMADKDIN	96		
	NTSSMADKDIN	4		
	NTSNMADKNIN	3		
	NTSXMADKDIN	3		
	NTANMADKDIN	2		
	NTSNMAGKDIN	2		
	NTSNMADKDVN	2		
	NTSBMADKDIN	2		
	NTSNLADKDIN	2		
	NTSYMADKDIN	1		
	NTSNIADKDIN	1		
	NTSNMADKDIT	1		
	NTSNMADKDSN	1		
	NTSNMADKDIK	1		
	NTXNMADKDIN	1		
	STSNMADKDIN	1		
	DTSDMADKDIN	1		
TOP 2	HNTLDKPTVDGWLTQINLNI		7240	
	VTAEMADKDIG	4623		
	VTAEMADKNIG	2515		escape
	VTAEMADKDIE	50		
	VTAEMADKDVG	20		
	VTAEMGDKDIG	11		
	VTAEMADKNIR	3		
	VTAELADKDIG	3		
	VTAEMADKDIR	2		
	VTAEMADEDIG	2		
	XTAEMADKDIG	2		
	VTAEMADQDIG	2		
	VTAEMADKSIG	1		
	VTAEMADKNIE	1		
	VTAEMADRDIG	1		
	VTAEMABKDIG	1		
	VTAVMADKDIE	1		
	VTAEMXDKNIG	1		
	VTAEMADKDJG	1		
TOP 3	HHQISMPTVDGWKTQITVNI		5399	
	NTADMADEDVN	4475		neutralized
	NTADMADEDIN	799		
	NTADMADENVN	27		
	NTANMADEDVN	18		
	NTADMADKDIN	14		
	NTADLADEDIN	10		
	NTANMADEDIN	7		
	NTADMADKDVN	4		
	NTADIADEDVN	3		
	NTADMADEDIS	3		
	NTADMADGDVN	3		
	NAADMADEDVN	2		
	DTADMADEDVN	2		
	NTADMADENIN	2		
	NTSDMADEDVN	2		
	NSADMADEDIN	2		
	NTADMADEGVN	2		
	NTADMAHEDAN	1		
	YTADMADEDVN	1		
	NTADMADDDVN	1		
	XTADMADEDVN	1		
	NTVDMADEDVN	1		
	NTGDMADEDVN	1		
	NTADMAAEDVN	1		neutralized
	NTADMABEDVN	1		
	NTTDMADEDIN	1		
	NTADMADEYVN	1		
	NTADMADEDVK	1		
	NTADMADADIN	1		
	NTTDMADEDVN	1		
	NTADMADEDVT	1		
	NIADMADEDVN	1		
	NTADVADEDVN	1		
	NTADMADEDAN	1		
	NTADMADEDIT	1		
	NTADMADEDFN	1		
	NTADMADEDLN	1		
	NTADMADEBVN	1		
	NTADMANEDVN	1		
	NTANMADDDIN	1		
	NTADMADVDVN	1		
TOP 4	HHVLSLPTVDGWQTQITVNI		3107	
	NTSNMADKNIN	2324		
	NTSNMADENIN	605		
	NTSNMADKDIN	66		
	NTSNMADQNIN	49		
	NTSNMADENVN	15		
	NTSNMADGNIN	7		
	NTSNMADENIT	7		
	NTANMADKDIN	6		
	NTSNMADKNVN	6		
	NTSNMADKNIX	4		
	NTSNMADKNIK	3		
	NTSNMADKSIN	3		
	NTSNMADKDVN	2		
	NTSNMADKNIB	2		
	NTSNMADRNIN	2		
	NTSDMADKNIN	2		
	NTSNMADKNID	2		
	NTSNEADENIN	1		
	NTFNMADKNIN	1		
TOP 5	HNTLDKPTIDGWLTQINLNI		1910	
	VTAEMADKDIG	1873		
	ITAEMADKDIG	9		
	VTAEMADKNIG	8		
	VTAEMADKDIR	7		
	VTAEMADKDIE	4		
	VTAEMADKDTG	3		
	VTAEMAYKDIG	2		
	VTAEMADRDIG	1		
	VTAEMANKDIG	1		
	VTAEMGDKDIG	1		
	VTAEMAXKDIG	1		
TOP 6	QHKLTLPVVAGWRTQITVNV		1373	
	TTAELADDDIS	1235		neutralized
	TTAELADEDIS	90		
	TTAELADDDIT	25		
	TAAELADDDIS	4		
	TTAELADGDIS	4		
	TTTELADEDIS	3		
	TAAELADVDIS	2		
	TTAELAGDDIS	2		
	TTVELADDDIS	1		
	TTAELADKDIS	1		
	TTAQLADDDIS	1		
	TTAELADDDVS	1		
	TTAELADDDIP	1		
	TTAELADDDTS	1		
	TTAELADYNIS	1		
	TTAELADDDLS	1		
TOP 7	THALSKPNIAGWLTQITLNS		1371	
	STFNMADKNIK	1360		
	STFNMADKNIQ	2		
	STFNMADGNIK	2		
	STFNMADKDIK	2		
	STFNMADKNVK	1		
	STFNMAGKNIK	1		
	STFSMADKNIK	1		
	STFNMADKNIN	1		

STFNMADKNIE 1

TOP 8 HHVLSLPTIDGWQTQITVNI 1359
NTSNMADKDIN 888 neutralized
NTSNMADKNIN 412 neutralized
NTSNMADRDIN 17
NTSNLADKDIN 16
NTSNMADENIN 15
NTSSMADKDIN 3
NTSNMADKDIS 2
NTSNMADRNIN 1
NTSNMVDKDIN 1
NTSTMADENIN 1
NTSDMADKDIN 1
NTSNMADGNVN 1
NTSNMADKNIT 1

TOP 9 HTQLTKPTIDGWLTQINLNI 987
VVAELADKDIG 967
VVAELADKNIG 6
VIAELADKDIG 5
VVAELADKDTG 2
VVAEMADKDIG 2
VVAELADKDVG 2
IVAELADKDIG 1
VVAELANKDIG 1
VAAELADKDIG 1

TOP 10 HHVLNKTTIDGWRTQITVNI 859
NTSEMADEDIN 686
NTSEMADEDIT 152
NTSELADEDIN 16
NTSEMADEDIS 3
NTSEMADKDIN 2

TOP 11 TYALSKPNIAGWLTQITLNS 785
STFNMADKNIK 784
STFNMADKSIK 1

TOP 12 HNTLDKPTVDGWLTQITLNI 719
VTAEMADKNIG 662
VTAEMADKTIG 28
VTAEMADRNIG 7
GTAEMADKNIG 4
VTAEMADRIIG 3
VSAEMADKNIG 2
VTAEMADRVIG 2
VTAEMADQNIG 2
VTAEMADENIG 2
VTAEMADKNIR 2
VTAEMADINIG 1
VTAEMAAQDIG 1
GTADMADKNIG 1
VTAEMADXNIG 1
VTAGMADKNIG 1

TOP 13 HNTTKLPTVDGWYTQITLNI 592
VTAEMADKDIG 582
VTAEMADKDVG 2
VIAEMADKDIG 2
VTAEMADKNIG 2
VTAEMADEDIG 2
VTAEMADKDMG 1
ITAEMADKDIG 1

TOP 14 HNTLDKPTMDGWLTQINLNI 572
VTAEMADKNIG 409
VTAEMADKDIG 160
VTAEMADKNIE 2
VTAEMADKBIG 1

TOP 15 HHVLSLPTIDGWLTQITVNI 560
NTSNMADKDIN 525
NTSNMADKNIN 28
NTSNMADKXIN 2
NTSNMADKDIT 2
NTSNMADKDVN 1
NTSNKADKDIN 1
NTSDMADKDIN 1

TOP 16 HNTTSLPTINGWYTQITLNI 537
VVAELADKDIG 535
VVAELADKGIG 2

TOP 17 HHVISLPTVDGWLTQITVNI 528
NTSNMADKDIN 528

TOP 18 HNTTSLPTIDGWYTQITLNI 467
VVAELADKDIG 464
VVAELANKDIG 1
VVXELADKDIG 1
VVAELADKDVG 1

TOP 19 HNTTNLPTIDGWYTQITLNI 465
VVAELADKDIG 319 escape
VVAELADKDVG 88
VVAELADKNIG 45
VFAELADKDIG 5
VVAEMADKDIG 2
EVAELADKNIG 1
VVADLADKDIG 1
VIAELADKDIG 1
VVAELADKDVR 1
VVAELADKDIR 1
AVAELADKNIG 1

TOP 20 HHVLNKTTIDGWKTQITVNI 458
NTSEMADEDIN 450
NTSEMADEDIT 3
NTSEMADEDIS 2
NTSELADKDIN 1
NTAEMADEDIN 1
NTSELADEDIN 1

TOP 21 HAQLTKPTIDGWLTQINLNI 337
VVAELADKDIG 334
VVAELADRDIG 2
IVAELADKDIG 1

TOP 22 HHVLSLPTIDGWRTQITVNI 253
NTSNMADKDIN 233
NTSNMADRDIN 6
NTSNMADENIN 5
NTSNMADKDVN 5
NTSSMADKDIN 2
NTANMADKDIN 1
NTSNMPDKDIN 1

TOP 23 LSVLNRSTINGWKTQITVNV 244
NTSELADEDIS 229 neutralized
NTSELADEDIF 6
NTSELADEDVS 4
NTSELADEDMS 2
NTSELADEDLS 1
NTSELADENIS 1
NTSELADEEIS 1

TOP 24 HHVLNLPTIDGWQTQISVNI 215
NTSNMADKDIN 210
NTSNMADKDVN 4
NTSNMADEDIN 1

TOP 25 QHKLTLPVVAGWRTQVTVNI 185
TTTELADEDIS 113
TTAELADENIS 31
TTAELADEDIS 21
TTTELADENIS 14
TTTELADKDIS 2
TTTELADEDIT 2
ATTELADEDIS 1
TKTELADEDIT 1

TOP 26 QQELSKPIVAGWRTQIQLNI 184
NTVELADDDMN 181
NTVELADGDMN 2
NTVELADDGMN 1

TOP 27 QHKLTLPIVAGWRTQVTVNV 165
TTAELADDDIS 123
TTAELADVDIS 37
TTAELADDDIA 3
TTAELADDDMS 1
TTAELADDDVS 1

TOP 28 HHKITLPTVDGWKTQITVNI 152
NTADMADEDIN 134
NTANMADEDIN 7
NTADMADADIN 6
NTADMADEDTN 4
NTADMADEDIT 1

TOP 29 HHQITLPTVDGWKTQITVNI 144
NTADMADEDIN 141
NTADMSDEDIN 1
NTADMADKDIN 1
NTANMADEDIN 1

TOP 30 HHVLNKTTVDGWKTQITVNI 136
NTSEMADEDIN 136

TOP 31 QQMLSKPVIDGWQTQITVNV 126
NTTEMADKDIN 122 neutralized
NTTEMADKNIN 2
NTTEMADKGIN 1
NTTEMADKEIN 1

TOP 32 HHKITLPTVDGWKTQFTVNI 117
NTADMADEDIN 109 neutralized
NTANMADEDIN 6
NTADMADENIN 2

TOP 33 HNTLDRPTIDGWLTQINLNI 112
VTAEMADKDIG 112

TOP 34 HHVLNKTTIDGWKTQVTVNI 111
NTSEMADENIN 101
NTSEMADEDIN 10

TOP 35 HHVLSLPTIDGWKTQITVNI 103
NTSNMADKDIN 102
NTSNMADRDIN 1

TOP 36 LSVLNKSTINGWKTQITVNV 98
NTSELADEDIS 98

TOP 37 HNTLEKPTVDGWLTQINLNI 94
VTAEMADKDIG 86
VTAEMADKDIE 4
VTAEMADKXIG 2
VTAEMADKNIG 2

TOP 38 HHKITLPTIDGWKTQITVNI 91
NTADMADEDIN 84
NTANMADEDIN 7

TOP 39 HNTTNLPTVDGWYTQITLNI 84
VVAELADKDIG 83
IVAELADKDIG 1

TOP 40 QHKLTLPIVAGWRTQITVNV 73
TTAELADDDIS 67
TTAELADYDIS 2
TTAELADEDIS 2
-TAELADDDIS 1
TTAELADDDIT 1

TOP 41 LSVLNKTTINGWKTQITINI 72
TTSDLADEDIT 48
NTSDLADADIT 15
NTSDLADANIT 6
NTSDLADVNIT 3

TOP 42 HNTTRLPTVDGWYTQITLNI 70
VTAEMADKDIG 62
VVAELADKDIG 6
VTAEMADKVIG 2

TOP 43 LSVLNKSTINGWRTQITVNV 70
NTSELADEDIS 70

TOP 44 HNTLDKPTVDGWLTQVTLNI 59
VTAEMADKNIG 58
VTAEMADKSIG 1

TOP 45 HHALSLPTVDGWLTQITVNI 57
NTSNMADKDIN 57

TOP 46 HHVLSLPTVDGWLTQFTVNI 56
NTSNMADKDIN 56

TOP 47 HNTLDKPTVDGWLTQINLNV 56
VTAEMADKDIG 56

TOP 48 HNTLNKPTIDGWLTQINLNI 54
VTAEMADKDIG 53
VTAEMGDKDIG 1

TOP 49 HHVLNLPTVDGWQTQITVNI 54
NTSNMADENIN 30
NTSNMADQNIN 10
NTSNMADKDIN 8
NTSNMADKNIN 4
NTSNMADGNIN 2

TOP 50 LSILNKTTINGWKTQITINI 53
TTSDLADEDIT 26
NTSDLADANIT 11
NTSDLADTNIT 7
NTSDLADVNIT 6
TTTDLADEDIT 3

TOP 51 HHQISMPTIDGWKTQITVNI 51
NTADMADEDVN 26
NTADMADEDIN 23
NTTDMADEDIN 1
NTANMADEDIN 1

TOP 52 HTQLNKPTIDGWLTQINLNI 48
VVAELADKDIG 48

TOP 53 HNTLDKPTVNGWLTQINLNI 46
VTAEMADKNIG 43
VTAEMADKSIG 2
VTAEMADKDIG 1

TOP 54 HHVLNLPTVDGWQTQISVNI 43
NTSNMADKDIN 42
NTSNMADKDVN 1

TOP 55 HHQISMPTVDGWRTQITVNI 35
NTADMADEDVN 21
NTANMADKDIN 7
NTADMADEDIN 3
NTADMADKDIN 2
STADMADEDVN 2

TOP 56 QHKLTLPVVAGWRTQITVNI 34
TTTELADEDIS 19
TTTDLADEDIT 5
TTAELADDDIS 4
TTAELADENIS 4
TTAELADEDIS 2

TOP 57 HHQISLPTVDGWKTQITVNI 33
NTADMADENIN 26
NTADMADEDVN 4
NTADMADEDIN 1
NTADMANENIN 1
NTADMADEKIN 1

TOP 58 HHQISMPTVDGWKTQITINI 32
NTADMADEDIN 23
NTADMADEDVN 6
NTVDMADEDIN 3

TOP 59 HHQISMPTVDGWKTQVTVNI 30
NTADMADEDVN 16
NTADMADEDIN 14

TOP 60 QHKLTLPVVAGWRTQVTVNV 29
TTAELADDDIS 22
TTTELADEDIS 5
TTAELADDGIS 1
TTAELADEDIS 1

TOP 61 HHILNKTTIDGWRTQITVNI 29
NTSEMADEDIN 21
NTSEMADENIN 8

TOP 62 QHKLTLPIVAGWRTQVTVNI 29
TTTELADDDIS 28
TTTELADEDIS 1

TOP 63 HNTLEKPTIDGWLTQINLNI 27
VTAEMADKDIG 27

TOP 64 HHQISMPTVDGWQTQITVNI 25
NTADMADEDVN 25 neutralized

TOP 65 HHVLNRTTVDGWRTQITVNI 24
NTSEMADEDIN 24

TOP 66 QHKLTLPVVAGWKTQITVNI 23
TTTELADGDIS 10
TTAELADGDIS 7
TTTELADEDIS 4
TTTELADKDIS 1
TTAELADGDIP 1

TOP 67 LSVLNRTTINGWKTQITINI 23
TTSDLADEDIT 21
TTSDLADVDIT 2

TOP 68 HHVLSLPTIDGWQTQISVNI 22

NTSNMADKDIN 21
NTSNMSHKDIN 1

TOP 69 HHKISMPTVDGWKTQITVNI 22
NTADMADEDVN 15
NTADMADEDIN 7

TOP 70 HHVLSLPTVDGWLTQITVNV 22
NTSNMADKDIN 22

TOP 71 HHVLSLPTMDGWQTQITVNI 21
NTSNMADKNIN 20
NTSNMADKDIN 1

TOP 72 HHVLNLPTIDGWKTQISVNI 19
NTSNMADKDIN 19

TOP 73 HNTLDKPTVDGWITQITLNI 19
VTAEMADKNIG 19

TOP 74 HNTLDKPIIDGWLTQINLNI 19
VTAEMADKDIG 19

TOP 75 HHVLSLPTVDGWLTQVTVNI 18
NTSNMADKDIN 12
NTSDMADKDIN 6

TOP 76 HTQLTKPTIDGWLTQINLNV 17
VVAELADKDIG 17

TOP 77 HNTLGKPTMDGWLTQINLNI 17
VTAEMADKNIG 17

TOP 78 HNTTPLPLIDGWYTQITLNI 15
VVAELADKDIG 15

TOP 79 HNTTSLPTVDGWYTQITLNI 15
VVAELADKDIG 14
SVAELADKDIG 1

TOP 80 HTQLTKPTIDGWLTQISLNI 15
VVAELADKDIG 15

TOP 81 HDTTNLPTIDGWYTQITLNI 15
VVAELADKNIG 15

TOP 82 LSILNRTTINGWKTQITINI 15
TTSDLADEDIT 12
TTSDLAEEDIT 3

TOP 83 HHVLSLPTVDGWLTQITINI 15
NTSNMADKDIN 15

TOP 84 LSVLSKSTINGWKTQITVNV 15
NTAELADEDIS 14
NTAELADEDIY 1

TOP 85 HHILNKTTIDGWKTQITVNI 15
NTSEMADEDIN 13
NTSEMADGDIN 2

TOP 86 HHVLNKTTIDGWRTQVTVNI 14
NTSEMADEDIN 13
NTSEMADENIN 1

TOP 87 THSLSKPNIAGWLTQITLNS 13
STFNMADKNIK 13

TOP 88 HTQLSKPTIDGWLTQINLNI 13
VVAELADKDIG 13

TOP 89 QQKLTLPIVAGWRTQVTVNV 13
TTAELADVDIS 8
TTAELADDDIS 5

TOP 90 HNTTPLPTIDGWYTQITLNI 12
VVAELADKDIG 12

TOP 91 QHQLTLPVVAGWRTQVTVNV 12
TTAELADDDIS 12

TOP 92 HSKLDKPTIDGWLTQINLNI 12
VVAELADKDIG 12

TOP 93 QHKLTLPIVSGWRTQVTVNV 11
TTAELADDDIS 10
TTAELADVDIS 1

TOP 94 HNTTNLPTINGWYTQITLNI 11
VVAELADKDIG 11

TOP 95 HNTTKLPTVDGWYTQVTLNI 11
VTAEMADKDIG 11

TOP 96 HHKITLPTVDGWKTQVTVNI 10
NTADMADEDIN 8
NTANMADEDIN 2

TOP 97 HNTLDRPTVDGWLTQINLNI 10
VTAEMADKDIG 10

TOP 98 HHVLNLPTIDGWQTQITVNI 10
NTSNMADKDIN 7
NTSNMADKNIN 3 neutralized

TOP 99 HHILNKTTIDGWKTQVTVNI 10
NTSEMADENIN 10

Table S2. The TOP 305 most frequent discontinuous peptides on 2D1 B-cell epitope region in HA dataset, with the variety of extended 2D1 B-cell epitope. There are 2,198 different discontinuous peptides on 2D1 B-cell epitope region (sorted by frequency) among 45,812 HA sequences. The TOP305 most frequent discontinuous peptides (≥10 strains with identical discontinuous peptides on B-cell epitope region) are listed here. For each discontinuous peptide, its surrounding discontinuous peptides were also listed with frequencies. The surrounding residues were defined as residues with a distance to antibody between (4Å, 6Å]. The discontinuous peptides identical to validated strains both on B-cell epitope and extended B-cell epitope regions are marked.

TOP 1	FPTSSPNDKGNSYPKSKSINET	10386	
	WHLYDYQYKA	10270	neutralized
	WHIYNYQYKA	35	
	WHLYNYQYKA	33	
	WHIYDYQYKA	10	
	WHFYDYQYKA	6	
	WHLYDYQNKA	5	
	WHLVDYQYKA	4	
	WHLYDYQYKT	4	
	WYLYDYQYKA	2	
	WHLYDNQYKA	2	
	WHLYDYQHKA	2	
	WHLYXYQYKA	2	
	WHLYDYQYKX	2	
	GHLYDYQYKA	1	
	WHVYDYQYKA	1	
	WHLYDYQYKP	1	
	WHLYDYHYKA	1	
	WHLFNYQYKA	1	
	WHIYTYQYKA	1	
	WQLYDYQYKA	1	
	WXLYDYQYKA	1	
	WHLYDYQYKS	1	
TOP 2	--SFN-TVLNFKYPANVTPQNT	2377	
	WGLMNYARIS	2349	
	WGLMNYARMS	6	
	WGLMNYAKIS	2	
	WGLVNYARVS	2	
	WGLMNYARIN	2	
	WGLMDYARIS	2	
	WELMNYARIS	2	
	WGLVNYARIS	2	
	WGLMNYTRIS	2	
	WGLMNYARXS	1	
	WGLMNXARIS	1	
	WGLMNYSRIS	1	
	WGLLNYARIS	1	
	WGLMNYARKS	1	
	WGLMNFARIS	1	
	WGLMKYARIS	1	
	WXLMNYARIS	1	
TOP 3	--SFN-TVLKFKYPANVTPQNT	2037	
	WGLMNYARIS	1972	
	WGLMNYARMS	19	
	WGLMNYTRIS	14	
	WGLMNYAKIS	10	
	WGLMNHARIS	4	
	WGLMDYARIS	4	
	WGLMNYSRIS	4	
	WGLMKYARIS	3	
	WGLMNYARVS	2	
	WGLMTYARIS	2	
	WGLMNXARIS	1	
	WGLKNYARIS	1	
	WGLMNYARTS	1	
TOP 4	FPESSPNTKNGLYPNSKSATEN	1743	
	WHLYNYHYTA	1407	
	WHLYNYRYTA	304	
	WHLYNYNYTA	9	
	WHLYNYHHTA	4	
	WHLYNYYYTA	4	
	WHLYDYRYTA	3	
	WHLYDYHYTA	3	
	WYLYNYHYTA	2	
	WHLYNYHYTT	2	
	WHLHNYHYTA	1	
	WHLYSYHYTA	1	
	WHLYNYLYTA	1	
	WHLYNYRHTA	1	
	WHLYXYHYTA	1	
TOP 5	--SFN-TVLKYKYPANVTPQNT	1230	
	WGLMNYARIS	1084	
	WGLMNYARIC	125	
	WGQMNYARIS	5	
	WGLMNYVRIS	3	
	WGLVNYARIS	2	
	WGLMNYARIN	2	
	WGLMNYARLS	2	
	WGLMNYARAS	2	
	WGLMNYARIF	1	
	WGLINYARIS	1	
	WGLMTYARIS	1	
	WGLMNFARIS	1	
	WELMNYARIS	1	
TOP 6	FP-TI--VKNNAYPIDAQTRRN	897	
	WNQYNYTTTS	877	
	WNQYDYTTTS	9	
	WNQYNYTPTS	4	
	WNQFNYTTTS	3	
	WNQYNYTATS	2	
	RNQYNYTTTS	1	
	WNQYNYATTS	1	
TOP 7	VIAENPGPKNDKNKTTNPTDTQ	814	
	AGALTYGQTY	779	
	AGELTYGQTY	26	
	AGALIYGQTY	2	
	AGALAYGQTY	2	
	AGALTYGQAY	2	
	AGALKYGQTY	2	
	AGALTYRQTY	1	
TOP 8	IP-SSSDEKDNAYPTKRSNNEN	671	
	WHIYTYQYAS	654	
	WHIYTYHYAS	9	
	WHIYTYQYSS	3	
	WHIYTYRYAS	2	
	WHIYTYQYXS	1	
	WHIYTYQYTS	1	
	WHIYTYQYVS	1	
TOP 9	--GFT-SIDNAAFPQTKSRSTN	659	
	YGMYPYGLTF	642	
	YGTYPYGLTF	13	
	YGMYSYGLTF	2	
	YGIYPYGLTF	1	
	YGMYPYGLAF	1	
TOP 10	--GFT-TVSGSAYPVNVTPQNN	652	
	WGLMNYVRVS	575	
	WGLMDYVRVS	41	
	WGLMNYVRIS	23	
	WGLMSYVRVS	9	
	WGLMTYVRVS	4	
TOP 11	--EFQ-NVDGNAYPLNLTVNNI	644	
	WTQKNYKRLT	600	
	WTQKNYKRIT	36	
	WTQKNYERLT	3	
	WTQKDYKRLT	1	
	WTQKSYKRLT	1	
	WAQKNYKRLT	1	
	WTQKNYKRLS	1	
	WAQKNYKRIT	1	
TOP 12	--GFTGSIKGQNFPQTNTRTSN	600	

YSTYTYGSNH 588
YSTYTYGSSH 3
YSTYKYGSNH 3
YTTYTYGSNH 2
YSTYTYGSND 1
YSTYTYGSDH 1
YPTYTYGSNH 1
YSTYTYGSHH 1

TOP 13 IP-SSSSEKNSTYPTKRSNNEN 497
WHIYTYQYAS 491
WHIYTYQYTS 5
WHIYTYXYAS 1

TOP 14 --GFT-TVSGSAYPLNVTPQNN 491
WGLMNYVRIS 456
WGLMDYVRIS 12
WGLMNYVRTS 12
WGLMNYVRVS 10
WGLMSYVRIS 1

TOP 15 IP-SSSDEKNNTYPTKRSNNEN 479
WHIYTYQYAS 476
WHIYTYQYTS 3

TOP 16 FPTSSPNDKGNSYPKNKSINET 478
WHIYNYQYKA 321
WHLYDYQYKA 152
WHLYNYQYKA 2
WHIYNYQYTA 1
RHLYDYQYKA 1
WHLYDYQXKA 1

TOP 17 FPESSPNTKNGLYPNSKSETEN 473
WHLYDYHYTA 404
WHLYDYRYTA 30
WHLYDYHYTT 23
WHLYNYHYTA 11
WHLYNYHYTT 3
WHLYDYQYTA 2

TOP 18 --SFD-TVLKYKYPANVTPQNT 468
WGLMNYARIS 462
WGLMNYAKIS 4
WGLINYARIS 1
WGLMNYTRIS 1

TOP 19 VIAEKPGPK-DNNKNTNPTDVQ 464
AGALAYGQTY 282
AGALTYGQTY 171
AGAQTYGQTY 5
AGALVYGQTY 2
AGALEYGQTY 2
AGALTYGQAY 1
AGTLAYGQTY 1

TOP 20 --GFT-SIDNAAFPQTKSKSSN 434
YGMYTYGLTF 347
YGTYTYGLTF 58
YGMYIYGLTF 12
YEMYTYGLTF 11
YGMYTYGLTS 1
YGMYPYGLTF 1
YGMYTYGRTF 1
YGIYTYGLTF 1
YGVYTYGLTF 1
YGLYTYGLTF 1

TOP 21 --GFT-TVSGSTYPVNVTPQNN 329
WGLMNYVRVS 262
WGLMNYVRIS 50
WGLMDYVRVS 5
WGLMNYVGVS 4
WGLMSYVRVS 3
WGLMNYVRES 2
WGLVNYVRVS 1
WGLMNYIRVS 1
LGLMNYVRIS 1

TOP 22 FP-STTGDKSAAYPVKGTNSEN 317
WVIYTYGYTS 300
WVVYTYGYTS 8
WVIYTYGYTX 2
WVIYTYGYTY 2
WVIFTYGYTS 2
WVIYNYGYTS 2
WVIFAYGYTS 1

TOP 23 IP-SSSNDKNNAYPTKRTNNEN 317
WHIYTYQYAS 304
WHIYTYQYTS 4
WHIYTYQYES 3
WHIYTYQYPS 1
WHVYTYQYAS 1
WHIYIYQYAS 1
WHINTYQYAS 1
WHIYXYQYAS 1
WHIYNYQYAS 1

TOP 24 FT-NGGANQS-TYPVRRTNKEN 309
WVIFTYKYAS 227
WVIFTYKYTS 47
WVVFTYKYAS 17
WVIFTYKYSS 16
WVIFTYKYES 1
WVIFTYKHSS 1

TOP 25 --SFN-TVLNYKYPENVTPQNT 309
WGQMNYVRIS 289
WGQMNYARIS 16
WGQMNYVRIX 2
WGQMNYVRIT 1
WGQMNYVKIS 1

TOP 26 VIAEKPGPK-DNYKNTNPTDVQ 304
AGALTYGQTY 232
AGALKYGQTY 52
AGAQTYGQTY 10
AGALAYGQTY 7
AGAITYGQTY 1
AGALTFGQTY 1
AGTLTYGQTY 1

TOP 27 --GFT-TVSGNAYPVNVTPQNN 299
WGLMNYVRVS 294
WELMNYVRVS 2
WGLMNYVRLS 2
WGLMNYVRIS 1

TOP 28 FPTSSPDDKGNSYPKSKSINET 275
WHLYDYQYKA 273
WHLYNYQYKA 2

TOP 29 --EFQ-SVDGNAYPLNLTINNI 254
WTQKNYKRLT 246
WTQKNYKRIT 7
WTQKNCKRLT 1

TOP 30 IP-SSSNDKNNAYPTKRSNNEN 251
WHIYTYQYAS 225
WHIYTYQYVS 20
WHIYTYKYAS 4
WHIYTYQYTS 2

TOP 31 IP-SSSDEKNSTYPTKKSNNEN 250
WHIYTYQYAS 248
WHIYAYQYAS 2

TOP 32 FPANSPNEKGTSYPKSKSTNET 245
WHLYNYQYTA 243
WHLYDYQYTA 1
WHLYNYQFTA 1

TOP 33 IP-DSSDEKDNAYPTKKGNNEN 224
WHIYTYQYAS 222
WHIYTYRYAS 1
RHIYTYQYAS 1

TOP 34 FP-STAGDKSAAYPVKGTNSEN 208
WVIYTYGYTS 200
WVIFTYGYTS 7
WVIYTYGYTX 1

TOP 35 VIAENPGPKNDNNKTTNSTDTQ 200
AGALTYGQTY 198
AGALAYGQTY 1
AGALTYGQAY 1

TOP 36 IP-SSPSEKNSTYPTKRSNNEN 198
WHIYTYQYAS 194
WHIYTYQYAT 4

TOP 37 --SFN-TVLNYKYPANVTPQNT 184
WGLMNYVRIS 74
WGLMNYIRIS 52
WGLMNYVGIS 14
WGLMNYVIIS 8

WGLMNYARIS 8
WGLMNYVIVS 8
WGLMNYIIIS 7
WGLMNYVKIS 6
WGLMNYIGIS 2
WGLMDYVRIS 2
WGLMNYVRMS 1
WGLMNYXRIS 1
WGLVNYIRIS 1

TOP 38 --DFN-TVLEYKYPANVTPRNT 180
WGLMNYVRIS 168
WGLMNYVGIS 5
WGLMNYVKIS 2
WGLMNYIRIS 2
WGLMSYVRIS 1
WGLMNYARIS 1
WGLMNYVXIS 1

TOP 39 --SFN-TVSKFKYPANVTPQNT 176
WGLMNYARIS 173
WGLMDYARIS 2
WGLMNHARIS 1

TOP 40 LP-DQTQTKGSNYPIKRSNNET 171
WHAYTYQYVS 166
WHAYTYQYIS 4
WHAYTYQYFS 1

TOP 41 FP-TI--VKNNAYPVDAQTRRN 161
WNQYNYTTTS 157
WNQHNYTTTS 2
WNQYDYTTTS 1
WNQYTYTTTS 1

TOP 42 LP-DRTQTKGSNYPVKGSNNET 160
WHAYTYQYTS 159
WHAYTYQYVS 1

TOP 43 IP-SSSDEKNNAYPTKRSNNEN 157
WHIYTYQYAS 150
WYIYTYQYAS 6
WHIYTYHYSS 1

TOP 44 FPGSSPNTKNGLYPNSMSVTEN 155
WHLYNYHYTA 152
WHLYNYHYSA 2
WHLYNYHFTA 1

TOP 45 FD-TK--VKSGQFPVTDEKNQN 152
WNQYTYKLTT 150
WNQYIYKLTT 1
WNQYTYKFTT 1

TOP 46 IP-SSSDEKNDAYPTKISNNEN 150
WHIYTYQYAS 133
WHIYTYQYTS 11
WHIYTYKYAS 6

TOP 47 FPATSPNDKGNSYPKSKSTNET 149
WHLYNYQYTA 145
WHLYNHQYTA 1
WHLYSYQYTA 1
WYLYNYQYTA 1
WHIYNYQYNA 1

TOP 48 FP-STAGDTTSSYPVKGTRSEN 147
WVIFTYGYTS 131
WVIFMYGYTS 8
WVIFSYGYTS 5
WVIFTHGYTS 2
WVLFTYGYTS 1

TOP 49 IP-SSSNEKNSAYPTKRSNNEN 144
WHIYTYQYAS 130
WHIYTYQYSS 12
WHIXTYQYAS 1
WHIYTYQYTS 1

TOP 50 --GFT-SIDNSAFPQTKARSTN 143
YGMYPYGLTF 143

TOP 51 --GFT-TVSGNSYPTNVTPQNN 136
WGLMNYIRIS 114
WGLMNYVRIS 13
WGLMNYVRVS 4
WDLMNYIRIS 2
WGLTNYIRIS 2
WGLMNYIRTS 1

TOP 52 FPESSPNTKNGLYPNSKSVTEN 130
WHLYNYHYTA 125
WHLYNYRYTA 3
WHVYNYHYTA 1
WHLYNYHHTA 1

TOP 53 IP-DSSDEKNDAYPTKKSNNEN 127
WHIYTYQYAS 127

TOP 54 --GFT-TVSGSSYPTNVTPQNN 127
WGLMNYIRIS 120
WGLMNYVRIS 3
WGLMNYVRVS 2
WGLMNYIRTS 2

TOP 55 IP-SSSDEKNDAYPTKRSNNEN 125
WHIYTYQYAS 124
WHIYTYQYTS 1

TOP 56 --GFN-TVLEYKYPANVTPQNT 125
WGLMNYVRIS 124
WGLMNYARIS 1

TOP 57 --DFN-TVSEYKYPANVTPRNT 119
WGLMNYVRIS 93
WGLMNYIRIS 24
WGLMNYIKIS 2

TOP 58 --EFQ-NVDGNAYPLNLTINNI 115
WTQKNYKRLT 112
WTQKNYKGLT 1
WTQKNYKSLT 1
WTQKNYERLT 1

TOP 59 FPTSSPNDKENSYPKSKSINET 113
WHLYDYQYKA 100
WHIYNYQYKA 7
WHLYNYQYKA 3
WHIYSYQYKA 2
WHLYDYXYKA 1

TOP 60 FP-STAGDRSAAYPVRGTNSEN 112
WVIYTYGYTS 112

TOP 61 FPASSPNEKGTSYPKSKSTNET 108
WHLYNYQYTA 107
WHFYNYQYTA 1

TOP 62 FPASSPNEKGNSYPKSKSVNET 107
WHLYNYQYTA 105
WHIYNYQYTA 2

TOP 63 IP-DSSDEKDNAYPTKKSNNEN 106
WHIYTYQYAS 106

TOP 64 IP-SSSDEKNSTYPTKRSNNEN 105
WHIYTYQYAS 105

TOP 65 FPASSPNEKGNSYPKSKSINET 100
WHLYNYQYTA 97
WHIYNYQYTA 2
WYLYNYQYTA 1

TOP 66 VIAEKPGPR-DNNKTTNPTDVQ 96
AGALTYGQTY 95
AGTLTYGQTY 1

TOP 67 FP-TI--VKNGLYPVDAQTRRN 93
WNQYNYTTTS 64
WNQFNYTTTS 15
WNQFNYTITS 5
WNQYNYTITS 4
WNQYNYTSTS 2
WNQSNYTTTS 1
WNQYNYITTS 1
WNQYNYXXTS 1

TOP 68 FP-STAGDKSSEYPVKGTNSEN 89
WVIFTYGYTS 89

TOP 69 FP-STTGDRSDPYSLKGTTIEN 87
WVIYTYGYTS 78
WVLYTYGYTS 9

TOP 70 --SFN-TVLDYKYPANVTPQNT 87
WGLMNYVRIS 77
WGLMNYIRIS 8

```
        WGLVNYVRIS     1
        WGLMNYVKIS     1

TOP 71  IP-NSSDEKDNAYPTKRSNNEN 86
        WHIYTYQYAS     85
        WHIYTYQQAS     1

TOP 72  --GFT-SIDNAAFPQTKARSTN 85
        YGMYPYGLTF     85

TOP 73  FT-NGGANQS-TYPVKRTNKEN 83
        WVIFTYKYSS     77
        WVIFIYKYSS     2
        WVIXTYKYSS     2
        WVIFTYKYAS     2

TOP 74  --EFQ-TVDGNAYPLNLTVNNI 81
        WTQKNYKRLT     71
        WTQKNYKRVT     3
        WTQKNYKRIT     3
        WTQKSYKRLT     2
        WTQKNYKGLT     1
        WTQKNYQRLT     1

TOP 75  FP-SI--VKNNNYPVDAQTRRN 80
        WNQYNYTTTS     73
        WNQYNYTITS     6
        WNQYSYTTTS     1

TOP 76  IP-SSSDEKNSAYPTKRSNNEN 79
        WHIYTYQYAS     79

TOP 77  --GFN-TVSGSTYPVNVTPQNN 79
        WGQMNYVKIS     60
        WGLMNYVRIS     15
        WGLMKYVRIS     2
        WGQMNYVKVS     1
        WGLMSYVKIS     1

TOP 78  FPANSPNEKGNSYPKSKSTNET 74
        WHLYNYQYTA     74

TOP 79  --SFN-AVLNFKYPANVTPQNT 73
        WGLMNYARIS     73

TOP 80  --EFQ-SVDGNAYPLNLTVNNI 72
        WTQKNYKRLT     71
        WTQKSYKRLT     1

TOP 81  FPESSPKNKNGSYPNSKSVKEN 70
        WHLYNYRYTA     70

TOP 82  FPANSPNDKGNSYPKSKSINET 70
        WHLYNYQYTA     68
        WHLYNYQYKA     2

TOP 83  FPESSPNTKNGLYPNSKSAKEN 68
        WHLYNYHYTA     66
        WHLFNYHYTA     2

TOP 84  --SFD-TVLNYKYPANVTPQNT 68
        WGLMNYVRIS     68

TOP 85  FP-STQGDKTAEYPVKGTNSEN 68
        WVIYTYGYTS     67
        WVIYTYGYAS     1

TOP 86  IP-SSSDEKNNTYPTKKSNNEN 67
        WHIYTYQYAS     67

TOP 87  FPATSPNEKGNSYPKSKSTNET 64
        WHLYNYQYTA     51
        WHLYSYQYTA     8
        WHLYNSQYTA     2
        WHLYTYQYTA     2
        WHLYRYQYTA     1

TOP 88  --SFN-TVLKYRYPANVTPQNT 62
        WGLMNYARIS     59
        WGLMNYTRIS     3

TOP 89  VIAEKPGPKNDNNKTTNSTDTQ 61
        AGALTYGQTY     59
        AGALTYGQPY     1
        AGALTYGQAY     1

TOP 90  FP-TI--VKSNSYPVDAQTKRN 61
        WNQYNYKTTS     61

TOP 91  IP-SSSNDKNSAYPTKRSNNEN 59
        WHIYTYQYAS     56
        WHIYSYQYAS     3

TOP 92  FPTSSPNEKGNSYPKSKSVNET 59
        WHLYNYQYTA     55
        WYLYNYQYTA     3
        WHLYDYQYKA     1

TOP 93  --EFQ-NVSGNAYPLNLTVNNI 58
        WTQKNYKRLT     57
        WTQKNYERLT     1

TOP 94  FPESSPNTKNGLYPNSKSTTEN 57
        WHLYKYHYTA     41
        WHLYNYHYTA     7
        WHLYDYHYTA     7
        WHLYNYRYTA     1
        WYLYKYHYTA     1

TOP 95  --SFN-TVLEYKYPANVTPQNT 55
        WGLMNYVRIS     45
        WGLMNYARIS     4
        WGLMNYVKIS     3
        WGQMNYVRIS     3

TOP 96  IP-SSPDEKDNAYPTKKSHNEN 54
        WHIYTYQYAS     51
        WHIYIYQYAS     3

TOP 97  IP-SSSNEKNSTYPTKRSNNEN 53
        WHIYTYQYAS     52
        WHIYTYQYTS     1

TOP 98  FPASSPNEKGNSYPKSKSTNET 53
        WHLYNYQYTA     52
        WHLYSYQYTA     1

TOP 99  --DFN-TVSDYKYPANVTPRNT 52
        WGLMNYVRIS     49
        WGLMNYIRIS     3

TOP 100 FD-SK--VKKDTYDFEGSVNRN 52
        WNNYNYKLTT     52

TOP 101 FD-SR--VKKDTYDFEGSVNRN 50
        WNNYNYKLTT     50

TOP 102 IP-SSPDEKNNTYPTKESHNEN 50
        WHIYTYKYTS     50

TOP 103 FPTNSPNDKGNSYPKSKSINET 50
        WHLYDYQYKA     44
        WHLYNYQYTA     3
        WHLYNYQYKA     3

TOP 104 FPESSPNTKNGLYPNSRSATEN 48
        WHLYNYHYTA     48

TOP 105 FPASSPNEKGNTYPKNKSVNET 48
        WHLYNYQYTA     46
        WHLYNYQYXA     1
        WHLYNYQSTA     1

TOP 106 FP-TI--VKDNAYPIDAQTRRN 48
        WNQYNYTTTS     47
        SNQYNYTTTS     1

TOP 107 FP-STVGDSSSAYPVKGTRSEN 47
        WVIFTYGYTS     47

TOP 108 --GFT-AVSGNTYPVNVTPQNN 46
        WGLMNYIRVS     38
        WGLMNYIRIS     7
        WELMNYIRVS     1

TOP 109 --NFN-TVLEYKYPANVTPRNT 46
        WGLMNYVKIS     37
        WGLMNYVRIS     9

TOP 110 FPTSSPNEKGNSYPKSKSINET 46
        WHLYDYQYKA     30
        WHLYNYQYKA     14
        WHLYNYQYTA     2

TOP 111 FP-STTGDRSDPYSLKGTTLEN 46
        WVVYTYGYTS     42
        WVIYTYGYTS     4
```

TOP 112 --SFN-TVLRFKYPANVTPQNT 42
WGLMNYARIS 42

TOP 113 --GFT-TVSGNAYPLNVTPQNN 42
WGLMNYVRIS 32
WGLMNYVRVS 9
WGLMDYVRIS 1

TOP 114 FP-TI--VKNGVYPVDAQTRRN 41
WNQYNYTTTS 40
WNQYNYTITS 1

TOP 115 FPESSPNTKSGLYPNSKSATEN 41
WHLYNYHYTA 41

TOP 116 FPTISPNDKGNSYPKSKSINET 41
WHLYDYQYKA 41

TOP 117 FP-STAGDKSSEYPVKGTRSEN 40
WVIFTYGYTS 36
WVIFMYGYTS 3
WVIFIYGYTS 1

TOP 118 FP-TI--VKNGLYPIDAQTRRN 40
WNQYNYTTTS 40

TOP 119 FPAISPNEKGNSYPKSKSVNET 40
WHLYNYQYTA 40

TOP 120 --EFQ-NVDGDAYPLNLTVNNI 39
WTQKNYKRLT 38
WTQKNYKRIT 1

TOP 121 IP-NSSDEKNNAYPTKKSNNEN 39
WHIYTYQYAS 33
WHIYTYQYVS 6

TOP 122 IP-DSSEEKDNAYPTKKGNNEN 38
WHIYTYQYAS 38

TOP 123 VIAEDPGPKNDKNKTTNPTDTQ 38
AGALTYGQTY 38

TOP 124 --GFN-AVSGNAYPVNVTPQNN 38
WGLMNYVRVS 34
WGLMNYIRVS 3
WGLMNYVRIS 1

TOP 125 IP-SSSNDKNSTYPTKRSNNEN 38
WHIYTYQYAS 35
WHIYTYQYTS 2
WHLYTYQYAS 1

TOP 126 FP-STQGDKTAEYPVNGTNSEN 38
WVIYTYGYTS 38

TOP 127 FP-TI--VKSGSYPVDAQTRRN 38
WNQYNYTTTS 19
WNQYNYITTS 18
WNQYDYITTS 1

TOP 128 FP-RT--VKSNSYPIDAQTKRN 37
WNQYDYKTTS 35
WKQYDYKTTS 2

TOP 129 FPSNSPNEKGNSYPKSKSTNET 37
WHIYNYQYTA 37

TOP 130 FP-TI--VKSGSYPVDAQTNRN 36
WNQYNYITTS 36

TOP 131 FD-TK--VKSEQFPVTDEKNQN 35
WNQYTYKLTT 33
WNQYTYRLTT 2

TOP 132 FPTSSPNDKGDSYPKSKSINET 35
WHLYDYQYKA 32
WHIYNYQYKA 2
WHLYNYQYKA 1

TOP 133 FPSSSPNEKENSYPKSKSINET 35
WHLYNYQYTA 35

TOP 134 FPERSPKNKNGSYPNSKSVKEN 33
WHLYNYRYTA 32
WHLYNYQYTA 1

TOP 135 --SFN-TVLNFKYSANVTPQNT 33
WGLMNYARIS 31
WGLMKYARIS 1
WGLINYARIS 1

TOP 136 --GFT-SIDNATFPQTKSKSSN 33
YGMYTYGLTF 24
YGLYTYGLTF 5
YGVYTYGLTF 3
YGMYIYGLTF 1

TOP 137 FPTSSPNDKGNSYPKSTSINET 33
WHLYDYQYKA 33

TOP 138 FP-STTGDKSAAYPVKGTKSEN 33
WVIYTYGYTS 31
WVIFTYGYTS 2

TOP 139 FPTNSPNEKGTSYPKSKSTNET 32
WHLYNYQYTA 32

TOP 140 IP-SSPNEKNSTYPTKRSNNEN 32
WHIYTYQYAS 32

TOP 141 FP-TI--VKSNSYPFDAQTKRN 31
WNQYNYKTTS 26
WNQYNYKATS 3
WNQYTYKTTS 2

TOP 142 FT-NGGANQS-TYPVQRTNKEN 31
WVIFTYKYAS 26
WVIFTYKYTS 5

TOP 143 FPTSSPNDKGNSYPKSNSINET 31
WHLYDYQYKA 26 escape
WHIYDYQYKA 5

TOP 144 FPENSPNTKNGLYPNSKSKTEN 31
WHLYDYHYTA 29
WHLYDYHYTT 2

TOP 145 FPESSPNNKEGSYPKKNSVNEN 31
WHLYKYQYTA 28
WHLYKYRYTA 2
WHQYKYQYTA 1

TOP 146 FPESSPNTKNGLYPNSKSKTEN 30
WHLYDYHYTA 26
WHLYDYRYTA 2
WHLYDYHYTT 1
WHLYDYHHTA 1

TOP 147 --EFQ-GVDGNAYPLNLTINNI 30
WTQKNYKRLT 29
WTQKNYKRIT 1

TOP 148 FPANSPNDKENSYPKSKSINET 30
WHLYNYQYTA 25
WHLYNYQYKA 4
WHIYNYQYTA 1

TOP 149 FP-STAGDTTSGYPVKGTTSEN 30
WVIYTYGYTS 30

TOP 150 FP-TI--VKNGNYPVDAQTRRN 28
WNQYTYTTTS 27
WNQYTYTATS 1

TOP 151 FPASSPNDKGNSYPKSKSINET 28
WHLYDYQYKA 27
WHLYNYQYKA 1

TOP 152 FPTTSPNDKGNSYPKRKSTNET 28
WHLYNYQYTA 28

TOP 153 IP-SSSNEKDNAYPTKISNNEN 27
WHIYTYQYAS 27

TOP 154 --DFN-TVSGNTYPMNVTPQNN 27
WGLMSYIKIS 11
WGLMSYVKIS 11
WGLMSYVKVS 3
WGLMSYVKTS 2

TOP 155 FP-STAGDESAAYPVKGTNSEN 27
WVIYTYGYTS 27

TOP 156 FPTSSPNDKGNSYPKNQSINET 26
WHIYNYQYKA 25

```
        WHLYDYQYKA     1

TOP 157 FP-TI--VKSNAYPIDAQTRRN 26
        WNQYNYTTTS     26

TOP 158 FPESSPNTKNGLYPNSKSAKES 26
        WHLYNYHYTA     26

TOP 159 --SFN-TVLNFKYQANVTPQNT 26
        WGLMNYARIS     26

TOP 160 --GFT-SIDNAAFPQTKSRSSN 25
        YGMYTYGLTF     24
        YGTYSYGLTF     1

TOP 161 FP-TI--VKDNAYPIDAQTRRD 25
        WNQYNYTTTS     25

TOP 162 IP-SSSDEKNNAYPTKISNNEN 25
        WHIYTYQYAS     24
        WHIYTYQYTS     1

TOP 163 --GFT-AVSGNAYPVNVTPQNN 25
        WGLMNYVRVS     15
        WGLMNYIRVS     8
        WGLMNYVSVS     1
        WGLMNYVRIS     1

TOP 164 VIAENPGPKNDKNKTTNSTDTQ 24
        AGALTYGQTY     24

TOP 165 --GFN-TVLEYKYPANVTPHNT 24
        WGLMNYVRIS     24

TOP 166 IP-SSSEEKNDAYPTKISNNEN 24
        WHIYTYQYAS     24

TOP 167 FPTSSPNDKXNSYPKSKSINET 24
        WHLYDYQYKA     21
        WHLYNYQYKA     3

TOP 168 FPDSSPNTKNGLYPNSKSVKEN 23
        WHLYKYHYTA     23

TOP 169 FP-TI--VKNNAYPTDAQTRRN 23
        WNQYNYTTTS     23

TOP 170 --GFN-TVSESTYPVNVTPQNN 23
        WGLMNYVRIS     13
        WGLMNYVRVS     10

TOP 171 FP-RI--VKSNSYPVDAQTKRN 23
        WNQYNYKTTS     23

TOP 172 IP-SSPDEKNNTYPTKKSHNEN 22
        WHIYTYKYTS     15
        WHIYIYKYTS     6
        WHIYTYKYAS     1

TOP 173 FPTSSPNEKENSYPKSKSVNET 22
        WHLYNYQYTA     21      escape
        WHIYNYQYTA     1

TOP 174 IP-SSSNEKNNAYPTKRSNNEN 22
        WHIYTYQYAS     22

TOP 175 --SFN-AVLKFKYPANVTPQNT 22
        WGLMNYARIS     22

TOP 176 --GFT-AVSRGAYPVNVSPQNN 21
        WGLRNYVRVS     21

TOP 177 --SFN-NVLNFKYPANVTPQNT 21
        WGLMNYARIS     21

TOP 178 IP-SSPDEKNNTYPTEKSHNEN 21
        WHIYTYKYTS     21

TOP 179 --SFN-AVLKYKYPANVTPQNT 21
        WGLMNYARIS     21

TOP 180 IP-SYSNEKNNTYPPKVNTNDN 21
        WHIYTYQHSS     14
        WHIYAYQYTS     4
        WHIYTYQSSS     2
        WHIYTYQYSS     1

TOP 181 IP-DSSDEKNNAYPTKKSNNEN 21
        WHIYTYQYAS     21

TOP 182 FPGSSPNTKNGLYPNSKSATEN 20
        WHLYNYHYTA     20

TOP 183 FPTSSPNDKENSYPKNKSINET 20
        WHIYNYQYKA     19
        WHLYDYQYKA     1

TOP 184 IP-TSGELKN-RYPVSKTNNEN 20
        WVIYTYVYMS     20

TOP 185 IP-SSSNEKDSAYPTKRSNNEN 20
        WHIYTYQYAS     20

TOP 186 VIAERPGPR-D-NKTTNPTDVQ 20
        AGALTYGQTY     20

TOP 187 IP-SSSNDKNNAYPTKMTNNEN 20
        WHIYTYQYAS     20

TOP 188 --GFT-TVDNGVFPQTKSKSSN 18
        YGMYTYGLTF     18

TOP 189 IA-TSGANRG-KYPVRGTNNEN 18
        WVIYTYIYSS     15
        WVIYTYVYSS     3

TOP 190 FPESSPNTKGGLYPNSKSETEN 18
        WHLYDYHYTA     18

TOP 191 --GFN-TVSESKYPVNVTPRNT 18
        WGLMNYVRIS     6
        WGLMNYVKIS     6
        WGLMNYIRIS     6

TOP 192 IP-DSSDEKNAAYPTKKSNNEN 18
        WHIYTYQYAS     18

TOP 193 FPISSPNDKGNSYPKSKSINET 18
        WHLYDYQYKA     18

TOP 194 FP-STAGDKSSEYPVKGTSSEN 17
        WVIFTYGYTS     17

TOP 195 FPESSPNTKNGLYPTSKSATEN 17
        WHLYNYHYTA     12
        WHLYNYRYTA     5

TOP 196 --GFT-NIDNAAFPQTKARNTN 17
        YGMYPYGLTF     17

TOP 197 IP-SSSDEKNSSYPTKRSNNEN 17
        WHIYTYQYAS     17

TOP 198 --GFT-TVSGSAYPENVTPQNN 17
        WGLMNYVRVS     17

TOP 199 IP-SSSDEKNDTYPTKRSNNEN 17
        WHIYTYQYAS     15
        WHIYTYRYAS     2

TOP 200 FP-SSSNNKDNVYKTKRNTNET 16
        WHLYTYQYSS     16

TOP 201 FPSSSPNEKGNSYPKSKSINET 16
        WHLYNYQYTA     16

TOP 202 --DFN-TVLEYKYPANVTPQST 16
        WGLMNYVRIS     16

TOP 203 --EFQ-SVDGDAYPLNLTVNNI 16
        WTQKNYKRLT     16

TOP 204 IP-NSSSEKNSAYPTKRSNNEN 16
        WHIYTYQYAS     16

TOP 205 --NFN-TVLNYKYPANVTPQNT 16
        WGLMNYIRIS     15
        WGLMNYVRIS     1

TOP 206 FPANSPNEKGSSYPKSKSTNET 16
        WHLYNYQYTA     16

TOP 207 FPESSPXTKNGLYPNSKSATEN 16
        WHLYNYHYTA     14
        WHLYNYRYTA     1
        WHLYNYNYTA     1
```

TOP 208 FPTSSPNDKGNSYPKSQSINET 16
WHLYDYQYKA 15
WHLYNYQYKA 1

TOP 209 --SFN-TVLNHKYPANVTPQNT 16
WGLMNYVIIS 10
WGLMNYVRIS 5
WGLMNYIRIS 1

TOP 210 --SFN-TVSESKYPVNVTPRNN 16
WGLMNYVRIS 16

TOP 211 FPTSSPNEKGNSYPKNKSVNET 16
WHLYNYQYTA 16

TOP 212 IP-SSPDEKNNTYPTKRSNNEN 15
WHIYTYQYAS 15

TOP 213 IP-SSSNYKNNAYPTKRTNNEN 15
WHIYTYQYAS 15

TOP 214 FPESSPKSKNGSYPNTMSMKEN 15
WHLYDYRYTA 15

TOP 215 IP-SSSSEKNSAYPTKRSNNEN 15
WHIYTYQYAS 15

TOP 216 --GFN-TVSESKYPANVTPRNT 15
WGLMNYVRIS 15

TOP 217 IP-SSPNEKNSAYPTKRSNNEN 15
WHIYTYQYAS 15

TOP 218 VIIEKPGPR-DNNKTTNPTDVQ 15
AGALTYGQTY 15

TOP 219 FP-STAGDESSEYPVKGTNSEN 14
WVIFTYGYTS 14

TOP 220 --GFT-TVSGNSYPINVTPQNN 14
WGLMNYIRIS 10
WGLMNYTRIS 3
WGLMNYIRTS 1

TOP 221 FPTSSPNDKGNSYPKSESINET 14
WHLYDYQYKA 14 escape

TOP 222 IP-SSSNDKDNAYPTKRTNNEN 14
WHIYTYQYAS 14

TOP 223 IP-SSSNDKDNAYPTKRSNNEN 14
WHIYTYQYAS 14

TOP 224 FP-TT--VKSGFYPVDAKTRRN 14
WNQYNYITTS 14

TOP 225 IP-SSPDEKDNAYPTKRSNNEN 14
WHIYTYQYAS 14

TOP 226 --GFT-TVSGNTYPVNVTPQNN 14
WGLMNYVRIS 7
WGLMNYVRVS 5
WGLMNYIRVS 2

TOP 227 IP-GSSNEKNNAYPTKRTNNEN 14
WHIYTYQYAS 14

TOP 228 FPESSPNTKNGSYPTSKSVTEN 14
WHLYNYHYTA 14

TOP 229 FP-STTGDKSAAYPVKGTSSEN 14
WVIYTYGYTS 14

TOP 230 FPESSPNNKNGLYPNSKSKTEN 14
WHLYNYHYTA 7
WHLYDYHYTA 5
WHLYDYNYTA 2

TOP 231 FP-STTGDRSAAYPVRGTNSEN 14
WVIYTYGYTS 14

TOP 232 FP-TI--VKNSAYPIDAQTRRN 14
WNQYNYTTTS 14

TOP 233 FPESSPNTKNGSYPNSKSVTEN 14
WHLYNYHYTA 14

TOP 234 --SFN-AVSKFKYPANVTPQNT 13
WGLMNYARIS 12
WGLMNHARIS 1

TOP 235 FP-AI--VKSNAYPIDAQTRRN 13
WNQYNYTTTS 13

TOP 236 FPATSPNDKENFYPKNNSTNET 13
WHLYNYRYTA 13

TOP 237 FPESSPKSKNGSYPNSKSVKEN 13
WHLYNYRYTA 10
WHLYDYRYTA 3

TOP 238 --NFN-TVLNFKYPANVTPQNT 13
WGLMNYARIS 12
WGLMDYARIS 1

TOP 239 IP-NSSDEKNNAYPTKRSNNEN 13
WHIYTYQYAS 13

TOP 240 IP-NSTNDKNNVYPTKRTNNET 13
WHIYTYQYTS 13

TOP 241 --GFTGSIKGQNFPQANTRTSN 13
YSTYTYGSNH 13

TOP 242 --GFT-SIDNATFPQTNSRSTN 13
YGMYPYGLTF 13

TOP 243 --EFQ-NVNGDAYPLNLTVNNI 13
WTQKNYKRLT 8
WTQKNYKRIT 5

TOP 244 FPESSPKTKNGLYPNSKSATEN 13
WHLYNYHYTA 12
WHLYNYRYTA 1

TOP 245 LP-DQTQTKGSNYPVKRSNNET 13
WHAYTYQYVS 13

TOP 246 FPESSPNTKNGLYPNSKSATES 12
WHLYNYHYTA 11
WHLYNYNYTA 1

TOP 247 FPTSSPNXKGNSYPKSKSINET 12
WHLYDYQYKA 12

TOP 248 FP-STAGDNSAAYPVKGTNSEN 12
WVIYTYGYTS 11
WVNYTYGYTS 1

TOP 249 FT-GGGNDKS-TYPVKRTNKEN 12
WVIFTYKYSS 12

TOP 250 FPTSSPNDKGNSYPKSKSINEN 12
WHLYDYQYKA 12

TOP 251 FPESSPNEKGNSYPKSKSVNET 12
WHLYNYQYTA 12

TOP 252 FPTSSPNDKGKSYPKSKSINET 12
WHLYDYQYKA 12

TOP 253 --GFT-SIDNAAFPQTKARNTN 12
YGMYPYGLTF 12

TOP 254 IP-SSANEKNNTYPTKRSNNEN 12
WHIYTYQYAS 12

TOP 255 --GFT-SMDNAAFPQTKSRSSN 12
YGTYPYGLTF 12

TOP 256 IP-SSSDEKDDAYPTKRSNNEN 12
WHIYTYQYAS 12

TOP 257 LP-DQTQTKGSNYPVQRSNNET 12
WHAYTYQYVS 12

TOP 258 --GFT-TVSGSSYSTNVTPQNN 12
WGLMNYIRIS 8
WGLMNYVRVS 4

TOP 259 --GFT-SIDNATFPQTKSKGSN 12
YGLYTYGLTF 12

TOP 260 LP-DRTQTKGSDYPVKGSNNET 12
WHAYTYQYTS 12

TOP 261 --QFT-NVNGNYGPINVTEVNI 12

WG-KTYKRSS 12

TOP 262 FPTSSPNDKGNLYPKNKSINET 11
WHIYNYQYKA 11

TOP 263 FPTSSPNDKGNSYPKSISINET 11
WHLYDYQYKA 11

TOP 264 FP-TI--VKSNAYPVDAQTKRN 11
WNQYNYKTTS 11

TOP 265 FP-STAGDKSAAYPVKGTSSEN 11
WVIYTYGYTS 7
WVIFTYGYTS 4

TOP 266 --GFIGSIKGQNFPRTNTRTSN 11
YSTYTYGSNH 11

TOP 267 IP-SSSNEKNNAYPTKRTNNEN 11
WHIYTYQYAS 7
WHVYTYQYAS 4

TOP 268 --RFT-SIDNAAFPQTKSKSSN 11
YGTYTYGLTF 7
YGMYTYGLTF 3
YGMYIYGLTF 1

TOP 269 FD-SR--VKKDTYDFEGTINRN 11
WNNYNYKLTT 11

TOP 270 FPANSPNEKGNSYPKSKSVNET 11
WHLYNYQYTA 11

TOP 271 --GFN-TVSEYKYPANVTPRNT 11
WGLMNYVRIS 11

TOP 272 FPENSPNTKNGLYPNSKSATEN 11
WHLYNYHYTA 10
WHLYNYRYTA 1

TOP 273 FPESSPNTKNGLYPNSKSTKEN 11
WHLYDYHYTA 11

TOP 274 FP-STAGDRSSAYPVKGTRSEN 11
WVIFTYGYTS 11

TOP 275 IP-SSSNDKNSSYPTKRSNNEN 11
WHIYTYQYAS 11

TOP 276 FP-TI--VKNNTYPIDAQTRRN 11
WNQYNYTTTS 11

TOP 277 --GFR-SINNQVFPQNQTRTTN 11
YGLYTYGLTF 11

TOP 278 FPESSPNTKNGLYPXSKSATEN 11
WHLYNYHYTA 10
WHLYNYRYTA 1

TOP 279 --GFT-NIDNAAFPQTKARSTN 11
YGMYPYGLTF 11

TOP 280 FPENSPNTKNGLYPNSKSETEN 11
WHLYDYHYTA 11

TOP 281 FPESSPKNKNGLYPNSKSETEN 11
WHLYNYHYTA 11

TOP 282 VIAEKPGPR-GNNKTTNPTDVQ 11
AGALTYGQTY 11

TOP 283 IP-NSSDEKNNTYPTKRSNNEN 11
WHIYTYQYAS 11

TOP 284 FPTSSPSDKGNSYPKSKSINET 10
WHLYDYQYKA 7
WHLYDYQYNA 2
WHIYNYQYRA 1

TOP 285 VIAEKPGPKNENNKTTNSTDTQ 10
AGALTYGQTY 10

TOP 286 FP-STRGDNSAQYPVSGTNSEN 10
WVIYTYGYTS 10

TOP 287 FPESSPNTKNGLYPNXKSATEN 10
WHLYNYHYTA 8
WHLYNYRYTA 2

TOP 288 FP-RI--VKSNSYPIDAQTKRN 10
WNQYDYKTTS 7
WNQYNYKTTS 3

TOP 289 FPESSPDNKNGLYPNSKSKKEN 10
WHLYEYNHTA 10

TOP 290 --NFN-TVLNYKYPAKVTPQNT 10
WGLMNYAIIS 10

TOP 291 IP-ASGELKN-RYPVSKTNNEN 10
WVIYTYVYMS 10

TOP 292 --GFT-NIDNVAFPQTKSKGSN 10
YGMYTYGLTF 10

TOP 293 --GFT-AVSGSTYPVNVTPQNN 10
WGLMNYVRVS 10

TOP 294 FPTSSPNEKGNSYPRNKSVNET 10
WHLYNYQYTA 10

TOP 295 --SFK-TVLNYKYPENVTPQNT 10
WGQMNYVRIS 10

TOP 296 --GFT-TVSGSTYPVNVSPQNN 10
WGLRNYVRVS 8
WGLMNYVRVS 2

TOP 297 LP-DRTQTKGSNYPVRGSNNET 10
WHAYTYQYTS 10

TOP 298 FPESSPNNKNGLYPNSKSTTEN 10
WHLYKYHYTA 8
WHLYNYHYTA 1
WHLYKYYYTA 1

TOP 299 VIAENPGPK-DNNKNTNPTDVQ 10
AGALTYGQTY 10

TOP 300 FP-AI--VKNGLYPIDAQTRRN 10
WNQYNYTTTS 10

TOP 301 LP-NETQTKGSNYPIKRSNNEN 10
WHAYTYQYTS 10

TOP 302 IP-NFSDEKNSTYPTKKSNNEN 10
WHIYTYQYAS 10

TOP 303 --GFT-AVSGNAYPLNVTPQNN 10
WGLMNYVRVS 10

TOP 304 FP-STAGDKSTAYPVKGTNSEN 10
WVIYTYGYTS 10

TOP 305 FPTSSPNDKGNSYPKSKSISET 10
WHLYDYQYKA 10
